# G protein-coupled receptors and inflammation resolution signaling networks in the heart: Pharmacology and potential for innovative therapeutics

**DOI:** 10.1016/j.pharmr.2026.100129

**Published:** 2026-02-28

**Authors:** Deanna K. Sosnowski, Terence E. Hébert, Dobromir Dobrev, Stanley Nattel

**Affiliations:** 1 Department of Pharmacology and Therapeutics, McGill University, Montréal, Québec, Canada; 2 Montreal Heart Institute Research Centre, Université de Montréal, Montréal, Québec, Canada; 3 Institute of Pharmacology, West German Heart and Vascular Center, University Duisburg-Essen, Essen, Germany; 4 Department of Integrative Physiology, Baylor College of Medicine, Houston, Texas; 5 Department of Pharmacology and Physiology, Faculty of Medicine, Université de Montréal, Montréal, Québec, Canada

## Abstract

Chronic, unresolved inflammation, and immune system activation contribute to the development and progression of human cardiovascular disease. Specialized proresolving mediators (SPMs), primarily the lipoxins and resolvins, demonstrate potent inflammation-resolving effects. SPMs bind to their respective G protein-coupled receptors (GPCRs) to transduce intracellular events and exert proresolving actions. Given the importance of immune cells in the cardiovascular system, the involvement of nonimmune cardiac cell-types in chronic cardiac inflammation, and the inflammation-resolving effects of SPMs, SPM-GPCR interactions may present efficacious new therapeutic targets for heart disease. In this review, we discuss the mechanisms potentially underlying these GPCR-mediated responses. We begin by providing a brief overview of SPM biosynthesis and the GPCRs implicated in SPM signaling. We then discuss literature demonstrating protective effects of SPMs in models of heart disease. We look in detail at the pharmacology of SPM-GPCR interactions, with a primary focus on formyl peptide receptor 2, GPR32, GPR18, and chemerin receptor 1. We then consider SPM-GPCR downstream signaling pathways in various cell models, including heterologous cell systems overexpressing human SPM-GPCR constructs, SPM-GPCR interactions in endogenous immune cells and effects in nonimmune cell types of the heart including cardiomyocytes, cardiac fibroblasts, endothelial cells, and vascular smooth muscle cells. We end by considering knowledge gaps and discussing future directions in SPM-GPCR interaction research. SPM-GPCR signaling networks and actions vary widely in different cell-types and disease contexts, and knowledge of the detailed pharmacology in the heart is quite limited, so extensive additional work on SPM-GPCR signaling is needed to capitalize on the rich therapeutic potential.

**Significance Statement:**

Inflammation resolution is a critical process in cardiac healing after injury or disease. Understanding of the pharmacology and G protein-coupled receptor-dependent signaling mechanisms of inflammation-resolving molecules in the heart is limited, heavily dependent on cell type, and sometimes conflicting. The knowledge gained about these signaling mechanisms in more basic cell systems can be used to facilitate future investigation of how these molecules work within the diverse cardiac cellular milieu as potential targeted therapeutics for heart disease.

## Graphical abstract

**Figure:**
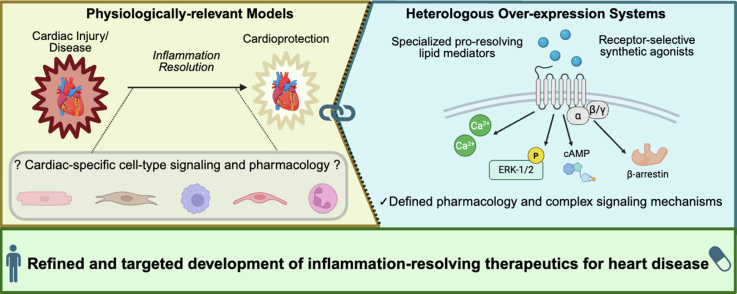

## I Introduction

Dysregulated inflammation and chronic immune-system activation underlie a plethora of chronic cardiovascular diseases (CVDs).^1^ The inflammatory response depends largely on the nature and intensity of the initial insult.^2^ We provide a brief summary of cardiac inflammation below, and refer readers to more detailed review articles in the area.^2^^,^^3^

**Fig. 1:**
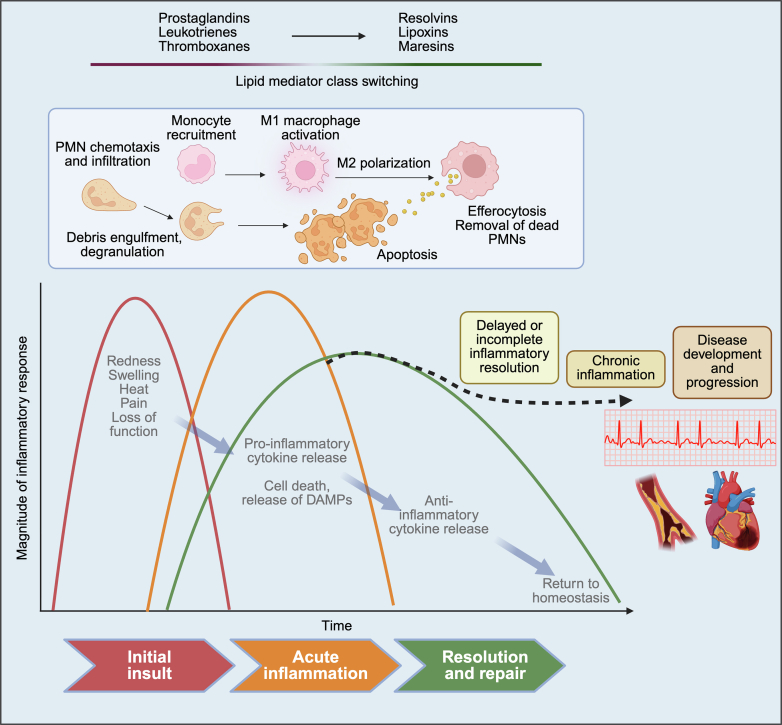
Activation of the innate immune system and the inflammatory response is a dynamic and active process. The initial inflammatory response is accompanied by PMN activation, proinflammatory lipid mediator production, and cytokine secretion. During the initial response, the active resolution process begins, characterized by production of SPMs, removal of apoptotic PMNs from the site of injury, and reparative macrophage polarization. If the resolution process is delayed or incomplete, this leads to a state of ongoing chronic inflammation, which promotes the development of chronic diseases, including CVD. DAMP, damage associated molecular pattern.

Inflammation and mobilization of the body’s immune response are dynamic processes (Fig. 1).^4^ Acutely damaged cardiac cells release damage associated molecular patterns, which alert the system to tissue injury.^4^^,^^5^ Neutrophils are recruited to the site of injury, proinflammatory cytokines are secreted, reactive oxygen species (ROS) released, and inflammatory lipid mediators produced.^4^^,^^5^ Once the immediate threat subsides, the body promotes tissue repair and a return to homeostasis.^4^^,^^5^ However, delayed or ineffective transition to this phase can prolong inflammation and cause chronic, low-grade immune-system activation, promoting the development of CVD.^4^^,^^5^

Attempts to directly target inflammation in CVD have produced modest outcomes in clinical trials.^6^, ^7^, ^8^ Some drugs currently used to treat CVD, such as statins, possess anti-inflammatory properties that might contribute to their efficacy.^9^ The GISSI-HF trial (NCT00336336) showed that daily administration of n-3 polyunsaturated fatty acid (PUFA) supplements provides a small protective effect against all-cause mortality and CVD hospital admissions in patients with heart failure (HF).^10^ In the REDUCE-IT trial (NCT01492361), patients with elevated triglyceride levels and established CVD received a daily dose of purified n-3 ethyl ester, icosapent ethyl, or placebo; the active agent reduced CVD-related deaths.^8^ However, many other large-scale randomized controlled trials of n-3 PUFAs for the primary or secondary prevention of CVD have shown little benefit.^9^^,^^11^, ^12^, ^13^, ^14^, ^15^ It has been suggested that any benefits of PUFA supplementation might be due to anti-inflammatory bioactive metabolites, rather than the parent molecules. Cytochrome P450 epoxygenase metabolites of the n-3 PUFAs, docosahexaenoic acid and eicosapentaenoic acid, have cardioprotective, anti-inflammatory, and antiapoptotic effects.^16^ Recently, another group of bioactive lipid mediators derived from n-3 and n-6 PUFAs have been highlighted—the specialized proresolving mediators (SPMs). Although the term SPM is generally used to connote lipid-mediator SPMs, a broad range of molecules with proresolving actions has been described, including peptides such as annexin A1 or lipocortin, small molecules such as adenosine, and large lipid molecules.^17^, ^18^, ^19^, ^20^ Four main classes of proresolving lipids have been identified, including lipoxins (Lx), resolvins (Rv), maresins (MaR), and protectins (PD)—these are the focus of this review.

SPMs have a unique set of properties; limiting cytokine production, reducing polymorphonuclear neutrophil (PMN) infiltration into damaged tissue, and promoting phagocyte action at the site of injury.^17^ Ultimately, this active “pro-resolution” process controls excessive inflammation, prevents tissue damage, and promotes repair, without detrimental immunosuppression. The discovery of SPMs has given rise to the novel research area of “resolution pharmacology,” addressing the basic underlying mechanisms, including SPM binding to G protein-coupled receptors (GPCRs) to activate intracellular signaling networks and exert their proresolving effects.^21^

Here, we aim to review the literature on resolvins, lipoxins, other specialized proresolving lipid mediators, along with synthetic proresolving molecules and peptides exogenously administered in models of heart disease, as well as studies investigating signaling mechanisms of these molecules via their respective GPCRs. We focus on heart disease (to the exclusion of vascular pharmacology), because the relevant literature on SPMs in CVD in general, including hypertension, atherosclerosis, and other forms of vascular disease, is too vast to cover within the context of this review. We begin by providing a brief overview of SPM biosynthesis and the GPCRs that have been implicated in SPM signaling. We then discuss the literature demonstrating protective effects of SPMs in models of heart disease, including acute myocardial infarction (MI), HF, atrial fibrillation (AF), and acute cardiac inflammatory disorders. We give particular emphasis to studies evaluating GPCR-dependency of SPM actions and include a section discussing the limited clinical evidence available. We then look in detail at the pharmacology of SPM-GPCR interactions, with a primary focus on formyl peptide receptor 2 (FPR2), GPR32, GPR18, and chemerin receptor 1 receptors. SPM-GPCR downstream signaling pathways are then compared in various cell models, beginning with established mechanisms in heterologous cell systems overexpressing human SPM-GPCR constructs and then moving on to SPM-GPCR interactions in endogenous immune cells and nonimmune cell types of the heart including cardiomyocytes, cardiac fibroblasts (cFBs), endothelial cells (ECs), and vascular smooth muscle cells (VSMCs). We end by considering knowledge gaps and discussing future directions in SPM-GPCR interaction research, including the potential development of receptor pathway-biased synthetic small molecules for heart disease therapy. A summary of our search strategy is provided in Supplemental Fig. S1.

## II Overview of specialized proresolving lipid-mediator biosynthesis and cognate G protein-coupled receptors

The details of SPM biosynthesis are complex, going beyond the scope of this review. We provide a brief overview here; the interested reader is referred to detailed reviews of SPM biosynthesis.^22^^,^^23^

### A N-3-derived specialized proresolving mediators

**Fig. 2:**
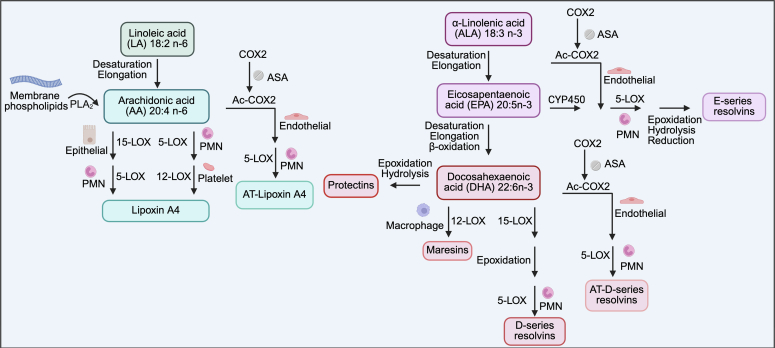
SPMs are biosynthesized from N-3 and N-6 PUFAs via a series of endogenous enzymatic reactions. SPM synthesis can occur in a transcellular manner involving multiple cell types. Aspirin-mediated acetylation of cyclooxygenase-2 (COX2) also promotes the formation of lipoxin A4 and D-series resolvins in their potent epimeric forms. ASA, acetylsalicylic acid; PLA_2_, phospholipase A_2_.

Humans obtain dietary n-3 PUFAs in the form of α-linolenic acid from sources such as fish, nuts, and seeds. α-Linolenic acid is metabolized by endogenous enzymes into a large family of bioactive lipids (Fig. 2).^24^, ^25^, ^26^ Myeloid cells are the primary source of these lipid mediators, however, they can also be generated by epithelial, EC, and VSMC in a stepwise manner through different cell types via transcellular biosysntheses.^27^^,^^28^ Elongation and desaturation reactions produce eicosapentaenoic acid, which gives rise to lipoxygenase (LOX)-derived E-series resolvins (RvE1–3).^5^^,^^16^ Further elongation, desaturation, and β-oxidation produce docosahexaenoic acid, from which LOX-isoforms and epoxidases produce the D-series resolvins (RvD1–6), maresins, and protectins.^5^ Recent studies have raised concerns about the reliable detection of endogenous SPMs in biological samples,^29^^,^^30^ questioning signal-to-noise ratios in SPM-corresponding peaks on chromatographs. There is, accordingly, an ongoing dispute about the biological role of endogenously produced SPMs. Our review focuses on the effects of exogenous administration of SPMs at therapeutically relevant doses, which are clearly effective in experimental models of heart disease.

### B N-6-derived specialized proresolving mediators

The n-6 PUFA linoleic acid is an essential fatty acid obtained in the diet from liquid vegetable oils present in large amounts in the Western diet.^16^ Arachidonic acid (AA) is produced via desaturation and elongation from linoleic acid. The body possesses ample quantities of AA, from which a range of proinflammatory lipid mediators, including the leukotrienes, prostaglandins, and thromboxanes, are produced under inflammatory or stressful conditions, as well as the SPM, lipoxin A4 (LxA4), via LOX and epoxide hydrolase (Fig. 2).^17^^,^^31^ Fine-tuning the biological switch regulating the production of proinflammatory mediators or SPMs in the same cell can allow for fine control of the inflammation resolution process in CVD. LxA4 undergoes a unique, transcellular mechanism of formation between epithelial cells, PMNs, and platelets.^32^ An intermediate metabolite is released from one cell and metabolized to the final product in a different cell type. However, the interaction between immune and nonimmune cell types is important for LxA4 synthesis, as well as its biological actions.^33^

### C Aspirin-triggered epimers

Potent epimers of endogenous SPMs can be generated through an alternative enzymatic pathway in vivo. Formation is triggered in the presence of aspirin via vascular endothelial cell cyclooxygenase-2 acetylation, followed by LOX oxygenation in PMNs (Fig. 2).^34^ This enzymatic sequence stereoselectively gives rise to the 15-*R* aspirin-triggered lipoxins (AT-Lx) and 17-*R* resolvins (AT-Rv).^34^ These epimers behave similarly to their nonaspirin-derived native counterparts, but have slower enzymatic deactivation.^5^ These compounds are important, because many patients with CVD receive aspirin, which might alter their endogenous SPM profiles.^35^

### D Specialized proresolving mediator receptors

**Fig. 3:**
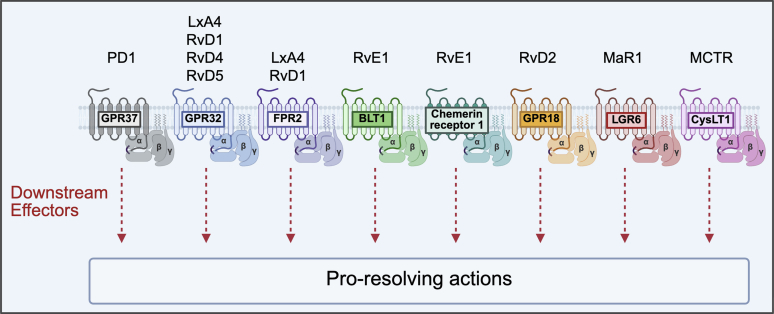
SPMs bind to their cognate GPCRs. Inflammatory resolution effects are transduced by activation of downstream signaling networks in a cell-type and physiological-context dependent manner. CysLT1, cysteinyl leukotriene receptor; LGR6, leucine-rich repeat-containing GPCR 6.

SPMs bind to GPCRs to exert their actions (Fig. 3).^21^ Changes in receptor conformation due to ligand binding cause the intracellular domains of the receptor to associate with G proteins. GTP hydrolysis dissociates G α- and G βγ-subunits, allowing them to independently modulate downstream effectors that mediate signal transduction and amplification.^36^^,^^37^ SPM-GPCRs were first identified on leukocytes, and later on epithelial cells, ECs, and VSMCs.^21^^,^^38^^,^^39^ These GPCRs are also present on cardiomyocytes (CMs) and cFBs, enabling these cells to respond to SPMs.^40^, ^41^, ^42^, ^43^ Whether CMs and cFBs respond to SPMs using the same set of intracellular signaling networks as leukocytes remains poorly understood.

Chemerin receptor 1, which binds RvE1, was originally identified on human blood-derived monocytes and macrophages, and is also present on neutrophils, dendritic cells, platelets, and T lymphocytes to a lesser degree,^38^^,^^44^^,^^45^ as well as the heart and vasculature.^38^ Rat VSMCs and murine cFBs express chemerin receptor 1 *Cmklr1* transcripts.^40^^,^^46^ Binding of RvE1 to chemerin receptor 1 promotes inflammation resolution by attenuating migration of neutrophils, enhancing macrophage phagocytosis, and reducing interleukin (IL)-12 secretion.^38^^,^^44^^,^^47^ RvE1 also binds to the leukotriene B_4_ receptor (BLT1) on human PMNs.^47^ RvE1 competes for binding with leukotriene B_4_ (LTB_4_) at BLT1 to attenuate proinflammatory LTB_4_ signals.^47^ Knockdown of BLT1 abolishes RvE1-mediated effects on PMN migration, which remain unaltered with chemerin receptor 1 knockdown, suggesting that anti-inflammatory effects of RvE1 on human PMNs may be primarily driven through binding to BLT1.^46^

The LxA4 receptor, FPR2, recognizes a diverse set of ligands ranging from peptides to lipids.^48^ FPR2 binds the endogenous proresolving protein annexin A1, as well as the SPM lipids, LxA4, and RvD1. Serum amyloid protein A binding transduces proinflammatory signals downstream of FPR2.^48^ It is thought that peptides and lipids may have distinct binding sites on FPR2, resulting in ligand-specific conformational changes of the receptor and transduction of distinct downstream signals.^49^^,^^50^ LxA4 promotes homodimerization of FPR2 to produce proresolution effects when expressed in human embryonic kidney (HEK) 293 cells, whereas formation of FPR2 heterodimers with other receptor isoforms, such as formyl peptide receptor 1 (FPR1), promotes apoptosis upon binding of proinflammatory ligands.^48^ Understanding signaling bias and how SPM-GPCRs elicit ligand-dependent effects is important in deciphering signaling mechanisms of SPMs.

Chemerin receptor 1, when activated by the endogenous proinflammatory peptide chemerin, induces strong chemoattractant effects on dendritic and natural killer cells, recruiting these cells into inflamed tissue to drive inflammation,^51^^,^^52^ but chemerin receptor 1 activation is also linked to proresolution actions.^38^ However, chemerin receptor 1 also possesses a dual function, with both proresolution and proinflammatory properties.^38^^,^^48^

Other GPCRs known to bind SPMs include GPR18, of which RvD2 is a ligand, as well as GPR32, which binds LxA4, RvD1, RvD3, and RvD5.^53^, ^54^, ^55^, ^56^ GPR32 has been detected on human phagocytic cells and VSMCs.^54^^,^^57^ The lack of a GPR32 homolog in mice complicates its study in murine models; however, some groups have engineered knock-in mouse models to express the human *GPR32* gene while simultaneously knocking out *Fpr2*.^57^ GPR18, originally known to bind endocannabinoid compounds and later identified as a receptor for RvD2, is expressed throughout the reproductive, central nervous, and immune systems. In the CV system, it has been detected on VSMC, ECs, and CMs in rats.

### E Synthetic specialized proresolving mediator analogs binding to specialized proresolving mediator-linked G protein-coupled receptors

The endogenous production of lipid mediators shows substantial variation in humans, heavily dependent on diet, medication, obesity, and age.^58^, ^59^, ^60^ Plasma concentrations of resolvins, lipoxins, their precursor lipids, as well as neutrophil FPR2 expression, are reduced in humans experiencing sleep disturbance, indicating how the balance of SPM formation, metabolism, and responsiveness depends heavily on individual characteristics.^61^^,^^62^ Furthermore, the different precursor lipids compete with each other for limited binding sites on enzymes catalyzing lipid mediator formation as well as for space within the plasma membrane of cells.^63^^,^^64^

A challenge with lipid-molecule therapeutics is that endogenously formed SPMs are often broken down nearly as quickly as they are formed.^65^ To overcome the labile nature of these molecules, synthetic peptides and small molecules with substantially improved stability and pharmacokinetic properties are being developed as specific agonists at FPR2, or as dual agonists at FPR1 and FPR2.^66^, ^67^, ^68^, ^69^ These synthetic analogs can also be biased toward specific downstream pathways.^70^ For example, 2 different synthetic compounds, Act-389949 and BMS-986235, activate G α_i/o_ to the same degree when binding to FPR2. However, Act-389949 more potently recruits β-arrestin, causing receptor internalization and a slower return of FPR2 to the cell surface.^70^ Therefore, BMS-986235 may have better efficacy if used for chronic therapy, because it induces less receptor desensitization. Studies examining the effects of these molecules in in vivo models of heart disease are detailed below.^68^^,^^71^^,^^72^

## III Specialized proresolving mediator-G protein-coupled receptor actions in models of heart disease

The effects of exogenous SPM and synthetic analog administration have been investigated in various models of cardiac disease. Much of the current literature focuses on agonists in murine and rat models of MI and ischemia-reperfusion (IR) injury. However, SPM effects are also demonstrated in other models of cardiac disease, including hypertensive and diabetic-induced HF, AF, and myocarditis. A detailed summary of the findings by disease model and drug compound is presented in Tables 1 and 2.^40^^,^^68^^,^^70^, ^71^, ^72^, ^73^, ^74^, ^75^, ^76^, ^77^, ^78^, ^79^, ^80^, ^81^, ^82^, ^83^, ^84^, ^85^, ^86^, ^87^, ^88^, ^89^, ^90^, ^91^, ^92^, ^93^, ^94^, ^95^, ^96^, ^97^, ^98^, ^99^, ^100^, ^101^

**Table 1:** In vivo studies demonstrating the effects of SPMs in models of cardiovascular disease

Disease Model	SPM Agonist	Experimental Model	SPM Effects	Receptor-Dependent Mechanism Reported?	References
Acute MI/IR injury	LxA4	♂ and ♀ New Zealand white rabbits Tracheal occlusion (5–9 min), then CPR LxA4 1.5 *μ*g/kg i.v. 30 s after restoration of circulation	Preserved MAP, LVSP, dP/dt ↓ cardiomyocyte apoptosis, serum cytokines, serum troponin I Preserved cardiac mitochondrial structural integrity	No	Chen et al 2013^73^
	LxA4	♂ Sprague-Dawley rats LAD ligation (30 min) Reperfusion (120 min) LxA4 100 *μ*g/kg i.v. preligation^*a*^ or LxA4 100 *μ*g/kg i.v. postreperfusion^*b*^ (30 min)	↓ duration VT and VF^*a*^ ↓ plasma inflammatory cytokine levels, increased plasma IL-10^*a,b*^ ↑ cardiac SOD and Na^+^/K^+^ ATPase activity^*a,b*^ Preserved cardiac mitochondrial ultrastructure^*a,b*^ Preserved Cx43 expression^*a,b*^	No	Zhao et al 2013^74^
	RvD1	♂ Sprague-Dawley rats RvD1 0.01,^*c*^ 0.1,^*d*^ or 0.3^*e*^*μ*g intraventricularly pretreatment LAD occlusion (40 min) Reperfusion (30 min–24 h)	↓ reduced infarct size, cardiac, caspase-3, and 8 activities^*d,e*^ ↓ neutrophil count^*c,d,e*^ ↑ Akt activation^*d,e*^ Cardioprotection abolished with Akt inhibitor	No	Gilbert et al 2015^75^
	RvD1	♂ C57BL/6 mice Permanent LAD ligation RvD1 3 *μ*g/kg s.c. post-MI (3 h), then daily for 1,^*f*^ 5,^*g*^ or 28^*h*^ days	Preserved cardiac function^*f,g*^ ↓ LV remodeling, CM hypertrophy^*g*^ ↑ cardiac neutrophil clearance^*f,g*^ ↑ reparative Lyc^*low*^ macrophages^*g*^ Normalized LV miR-transcription^*g*^ ↓ renal inflammation^*h*^	No	Halade et al 2018^76^
	RvD1	♂ C57BL/6 mice Permanent LAD ligation RvD1 3 *μ*g/kg s.c. post-MI (3 h), then daily for 1^*i*^ or 5^*j*^ days	↓ LV infarct size, fibrosis, M1 macrophage count, inflammatory, and fibrotic gene expression^*i,j*^ ↑ cardiac and splenic FPR2 expression^*i,j*^ ↑ LV and splenic neutrophil clearance^*j*^ ↑ splenic SPM synthesis^*j*^	No	Kain et al 2015^77^
	RvD1	♂ Sprague-Dawley rats RvD1 0.02,^*k*^ 0.1,^*l*^ or 0.3^*m*^*μ*g intraventricularly pretreatment LAD ligation (45 min) Reperfusion (180 min)	↓ infarct size, plasma CK-MB, LDH^*l,m*^↓ plasma and LV IL-6, TNF-α, MDA ↑ plasma SOD levels^*m*^ ↓ LV HMGB1, TLR4, NF-κ B expression^*m*^	No	Lui et al 2019^78^
	RvD1	♂ C57BL/6 mice LAD ligation (60 min) Reperfusion (7 days^*n*^ or 4 wk^*o*^) RvD1 in ROS-responsive liposome i.v.	↑ RvD1 cardiac accumulation, CD31^+^ cells, angiogenesis, efferocytosis, cardiac SPM production^*n*^ Preserved cardiac function^*o*^ ↓ infarct size, fibrosis^*o*^	No	Weng et al 2022^79^
	RvD1	♂ Wistar rats Permanent LAD ligation RvD1 2 *μ*g/kg i.p. daily for 29 days starting 1-day post-MI (early)^*p*^ or daily for 14 day starting 7 days post-MI (late)^*q*^	↓ AF inducibility^*p,q*^ Preserved atrial conduction velocity and cardiac function^*p,q*^ CD206^+^ atrial macrophage polarization^*p,q*^ ↑ atrial GPR32 levels^*p*^ ↓ NLRP3 inflammasome expression^*p,q*^	No	Hiram et al 2024^80^
	RvE1	♂ C57BL/6 mice Permanent LAD ligation RvE1 5 ng/g i.p. daily (days 1–7) post-MI^*r*^ or RvE1 5 ng/g i.p. daily (days 7–14) post-MI^*s*^	↓ CM apoptosis, Ly6C^+^ CD68^+^ macrophage infiltration, infarct size^*r*^ ↓ cardiac recovery, reparative Ly6C^*low*^ macrophage recruitment, proangiogenic markers^*s*^	No	Lui et al 2018^40^
Heart failure	RvD2	C57BL/6 mice TAC surgery RvD2 2 *μ*g/kg i.p. daily for 1 wk^*t*^ or 4 wk^*u*^ starting 1-day post-TAC	Preserved cardiac function^*u*^ ↓ ventricular remodeling^*u*^ ↓ Ly6C^*high*^ macrophages^*t,u*^ ↓ fibrosis and fibrotic gene expression^*u*^ ↓ CM hypertrophy^*u*^	Yes; protective effects abolished in GPR18^(−/−)^ mice and transplant of GPR18^(−/−)^ bone marrow into WT mice	Zheng et al 2024^81^
	LxA4	♂ ApoE^−/−^ mice STZ injection LxA4 5 *μ*g/kg i.p. twice weekly for 6 wk	↓ LV diastolic dysfunction and cardiac remodeling ↓ CD68^+^ iNOS^+^ LV macrophages ↑ CD206^+^ LV macrophages ↓ LV FPR2 gene expression ↓ LV inflammatory genes	No	Fu et al 2024^82^
Atrial fibrillation	RvD1	♂ Wistar rats MCT i.p. to induce right-heart disease RvD1 2 *μ*g/kg i.p. daily (21 days)	↓ AF, AFl inducibility, RA and LA fibrosis Preserved atrial conduction velocity Normalized ERP and APD M2 atrial macrophage polarization	No	Hiram et al 2021^83^
Myocarditis	AT-RvD1	C57BL/6 mice *Trypanosoma cruzi* infection i.p. AT-RvD1 5 *μ*g/kg i.p. daily for 20 days starting 40 days postinfection	↓ myocardial parasitic load, CM hypertrophy, LV TNF-α ↑ IL-10	Yes; protective effects abolished in FPR2^(−/−)^ mice	Carrillo et al 2021^84^
	RvD1	♂ C57BL/6 mice LPS i.p. RvD1 5 *μ*g/kg i.p. pretreatment	Preserved cardiac function ↓ CK-MB, CM apoptosis, LV cytokine gene expression, LV NF-κ B, MAPK activity M2 cardiac macrophage polarization	No	Wang et al 2020^85^
	RvD2	C57BL/6 mice CLP procedure or LPS i.p. RvD2 50–200 ng i.v. 6 h post-LPS	Preserved cardiac function ↓ BNP, cTnT ↓ ventricular CM death	No	Zhang et al 2024^86^
	RvE1	♂ C57BL/6 mice CLP procedure RvE1 1 *μ*g i.v. 1-h post-CLP	↓ cardiac dysfunction, p-ERK1/2, p-JNK, p-IKK, nuclear NF-κ B p65 ↑ cardiac p-Akt	No	Chen et al 2020^87^
	RvE1	♂ C57BL/6 mice Doxorubicin i.p. RvE1 2.5 *μ*g/kg i.p. pretreatment (30 min), then daily (5 days)	Preserved cardiac function, autophagic flux; beclin1, LC3II, p62 ↓ LDH, CK-MB, CM apoptosis, ROS MDA ↑ cardiac SOD, GSH activity, p-Akt	No	Zhang et al 2020^88^
	RvE1	♂ C57BL/6 mice aged 6–8 weeks LPS i.p. Resolvin E1 25 *μ*g/kg i.p. pretreatment (30 min)	Preserved cardiac function ↓ LDH, CK-MB, CM apoptosis, cardiac neutrophils, macrophages, LV NF-κ B, MAPK activity M2 CD206^+^ macrophage polarization	No	Zhang et al 2020^89^
	MCTR1	♂ C57BL/6 mice LPS i.p. MCTR1 0.15 nmol i.v. post-LPS (6 h)	Preserved cardiac function, mitochondrial ultrastructure, respiratory function ↓ cardiac cytokine expression ↑ mitochondrial biogenesis factors Sirt1, NRF1/2, TFAM	No	Yang et al 2020^90^

APD, action potential duration; BNP, *β*-type natriuretic peptide; CK-MB, creatinine kinase-MB; CLP, cecal ligation and puncture; CPR, cardiopulmonary resuscitation; cTnT, cardiac troponin T; Cx43, connexin 43; dP/dt, derivative of left ventricular pressure rise; ERP, effective refractory period; GSH, glutathione; HMGB1, high mobility group box protein 1; IKK, I κ B kinase; JNK, Jun N-terminal kinase; K^+^, potassium; LA, left atria; LAD, left anterior descending coronary artery; LC3, microtubule-associated protein 1A/1B-light chain 3; LDH, lactate dehydrogenase; LVSP, left ventricular systolic pressure; MAP, mean arterial pressure; MCT, monocrotaline; MDA, malondialdehyde; miR, microRNA; Na^+^, sodium; NRF1/2, nuclear respiratory factor 1/2; RA, right atria; Sirt1, sirtuin-1; SOD, superoxide dismutase; STZ, streptozotocin; TAC, transverse aortic constriction; TFAM, mitochondrial transcription factor A; TLR4, Toll-like receptor 4; VF, ventricular fibrillation; VT, ventricular tachycardia; WT, wild-type; ♂, male; ♀, female; ↑, increase; ↓, decrease; Δ, change.

Superscript letters specify the SPM effects associated with a particular SPM dose or treatment length in experimental designs with more than one SPM dose or time point tested.

**Table 2:** In vivo studies demonstrating the effects of recombinant and synthetic SPM-GPCR agonists in models of cardiovascular disease

Synthetic Agonist	Receptor	Disease Model	Experimental Model Details	Reported Effects	References
BMS-986235	FPR2	Acute MI	♂ C57BL/6 mice Permanent LAD ligation BMS-986235 0.3 mg/kg by mouth starting 4 days post-MI, then daily for 24 days	↓ LV infarct size, ↓ cardiac hypertrophy, ↓ LV dilation, preserved LVSP	Asahina et al 2020^72^
BMS-986235	FPR2	Acute MI	♂ C57BL/6 mice Permanent LAD ligation BMS-986235 3 mg/kg by mouth daily for 3 days^a^ or 0.3 mg/ kg^b^ or 3 mg/kg^*c*^ for 28 days starting 24 h post-MI	No Δ infarct size,^*a*^↓ LV infarct collagen,^*a*^↑ peri-infarct arginase-1 mRNA,^*a*^ no Δ total cardiac macrophage count,^*a*^↓ cardiac CD206^−^ macrophages and CD45^+^ neutrophils^*a*^ ↓ mortalilty,^*c*^↓ infarct size,^*c*^↑ infarct wall thickness,^*c*^↓ LV chamber area^*b*^	Garcia et al 2021^71^
BMS-986235	FPR2	Acute MI	♂ Sprague-Dawley rats Permanent LAD ligation BMS-986235 1 mg/kg by mouth daily for 5 days^*d*^ or 6 wk^*e*^ starting 48 h post-MI	Preserved EF,^*d*,*e*^↑ peri-infarct CD206^+^ macrophages,^*d*^↑ peri-infarct IL-10 levels,^*d*^↓ infarct size,^*e*^↓ LV remodeling^*e*^	Garcia et al 2021^71^
BMS-986235	FPR2	Acute MI	♂ C57BL/6 mice Permanent LAD ligation BMS-986235 1 mg/kg^*f*^ or 10 mg/kg^*g*^ by mouth daily for 3 days starting 24 h post-MI	↑ CD206^+^ cardiac macrophages^*f*,*g*^	Lupisella et al 2022^70^
BMS-986235	FPR2	Acute MI	♂ Sprague-Dawley rats Permanent LAD ligation BMS-986235 1 mg/kg^*f*^ or 10 mg/kg^*g*^ by mouth daily for 6 wk starting 48 h post-MI	Preserved EF and LV wall thickness^*f*,*g*^	Lupisella et al 2022^70^
BMS-986235	FPR2	IR injury	♂ Sprague-Dawley rats LAD ligation (60 min) Reperfusion (6 wk) BMS-986235 1 mg/kg by mouth daily for 6 wk	Preserved EF, ↑ viable myocardium, ↓ LV remodeling, preserved pressure-volume relationship	Garcia et al 2021^71^
BMS-986235	FPR2	Myocarditis	♂ C57BL/6 mice Hyper-homocysteine ad libitum for 3 wk in drinking water, then weekly arthritic serum injection i.p. and hyper-homocysteine ad libitum for an additional 3 wk BMS-986235 3 mg/kg by mouth daily for 2 wk starting 1 wk after first set of injections	Reduced LA remodeling, preserved E/A ratio, ↓ diastolic dysfunction ↓ cardiac neutrophil infiltration, ↓ cardiac monocyte-fibroblast number, ↓ proinflammatory fibroblast number No Δ CD45^+^ cell or cardiac cell FPR2 surface expression ↓ collagen fiber deposition	Margraf et al 2025^91^
RTP-026	FPR2	IR injury	♂ Sprague-Dawley rats LAD ligation (20 min) RTP-026 25,^*h*^ 50,^*i*^ or 500^*j*^*μ*g/kg i.v. prereperfusion Reperfusion (120 min) ♂ Wistar rats LAD ligation (30 min) Reperfusion (24 h) RTP-026 30 *μ*g/kg^*k*^ i.v. at 0, 3 and 6-h postreperfusion	↓ LV infarct size,^*h*,*i*,*j*^ no Δ MAP,^*h*,*i*,*j*^ ↓ neutrophil and classical monocyte cardiac recruitment,^*i*^ no effect on nonclassical monocytes^*i*^ No Δ circulating immune cell total counts,^*i*^↑ CD62L^+^ nonactivated blood neutrophils and monocytes,^*i*^↓ CD54^+^ blood leukocytes^*i*^ ↓ plasma IL-1 β,^*h*,*i*,*j*^ TNF-α,^*i*,*j*^ CXCL1^*i*^ ↓ plasma TxB2, PGE_2_ PGF_2_α^*i*^ ↓ LV infarct size,^*k*^↓ plasma troponin I^*k*^	Chen et al 2023^92^
Compound-43	FPR1/FPR2	Acute MI	♂ C57BL/6 mice Permanent LAD ligation Compound-43 10 mg/kg^*l*^ by mouth daily starting 24 h after surgery for 3 days *or* Compound-43 1 mg/kg^*m*^ or 10 mg/kg^*n*^ by mouth daily starting 24 h after surgery for 28 days	↑ macrophages expressing arginase-1 mRNA in peri-infarct zone,^*l*^↑ CD206^+^ cardiac macrophages,^*l*^ no Δ total cardiac macrophage count^*l*^ ↓ LV infarct size,^*m*,*n*^↓ LV structural remodeling,^*m*,*n*^ no Δ infarct collagen content,^*m*,*n*^ preserved LV pressure-volume relationship,^*m*,*n*^↓ scar strain^*m*,*n*^	Garcia et al 2019^93^
Compound-43	FPR1/FPR2	IR injury	♂ Sprague-Dawley rats LAD ligation (45 min) Reperfusion Compound-43 10 mg/kg by mouth daily for 6 wk starting 48 h post-MI	Preserved LVEDV and EF, preserved LV pressure-volume relationship, ↑ viable myocardium area	Garcia et al 2019^93^
Compound-43	FPR1/FPR2	IR injury	♂ C57BL/6 mice LAD ligation (40 min) Reperfusion (24 h^*o*^ or 7 days^*p*^) Compound-43 50 mg/kg i.p. daily starting before reperfusion ♂ C57BL/6 mice LAD ligation (60 min) Reperfusion (48 h) Compound-43 50 mg/kg i.p. daily starting before reperfusion	No attenuation of infarct size,^*o*^ AAR,^*o*^ or plasma cTnI levels,^*o*^ no attenuation of LV cell death,^*p*^ no Δ LV collagen deposition^*p*^ No Δ LV neutrophil or macrophage count, no reduction in CM death, ↑ circulating monocye and lymphocyte count	Qin et al 2017^68^
WKYMVM	FPR2	IR injury	♂ C57BL/6 mice LAD ligation (30 min) Reperfusion (60 min) WKYMVM 0.15 mg/kg^*q*^ or 0.3 mg/kg^*r*^ i.v. postreperfusion	↓ infarct AAR^*q*,*r*^	Gavins et al 2005^94^
WKYMVM	FPR2	Acute MI	♂ C57BL/6 mice Permanent LAD ligation WKYMVM 2.5 mg/kg i.p. daily for 4 days	↑ CAC mobilization into peripheral blood and heart, ↓ LV infarct size, ↓ CM apoptosis, ↓ fibrosis, preserved cardiac function, ↑ cardiac neovascularization	Heo et al 2017^95^
Ac_2-26_	FPR1/FPR2	IR injury	♂ C57BL/6 mice LAD ligation (30 min) Reperfusion (60 min) Ac_2-26_ 0.3 mg/kg^*s*^ or 1 mg/kg^*t*^ i.v. postreperfusion	↓ LV infarct size,^*t*^ preserved myofiber organization,^*t*^↓ mitochondrial swelling,^*t*^↓ cardiac neutrophil infiltration,^*t*^↓ LV chemokine levels^*t*^	Gavins et al 2005^94^
Ac_2-26_	FPR1/FPR2	IR injury	♂ Sprague-Dawley rats LAD ligation (25 min) Reperfusion (2 h) Ac_2-26_ 0.5 mg/kg i.v. 0 minutes postreperfusion^*u*^ or 1 mg/kg i.v. 0,^*v*^ 30,^*w*^ or 60^*x*^ minutes postreperfusion	↓ LV infarct size,^*u*,*v*,*w*,*x*^↓ LV dilation,^*u*,*v*^↓ cardiac IL-1 β levels,^*v*^↓ cardiac MPO levels,^*v*^↑ endogenous cardiac neutrophil and CM annexin A1 levels^*v*^	La et al 2001^96^
Ac_2-26_	FPR1/FPR2	IR injury	♂ Sprague-Dawley rats *Ex vivo* global no-flow ischemia (30 min) Reperfusion (30 min) Ac_2-26_ 0.3 μM at onset of reperfusion ♂ and ♀ C57BL/6 x 129/Svj mice *Ex vivo* global no-flow ischemia (22.5 min) Reperfusion (40 min) Ac_2-26_ 0.3 μM at onset of reperfusion	Recovered LVDP by 10 min postreperfusion, preserved LV ±dP/dt and LV RPP, ↓ cardiac LDH and CK release, ↑ p-Akt and p-PLB Preserved LVDP, LV ±dP/dt, and LV RPP 20 minutes postreperfusion, ↑ p-Akt	Qin et al 2013^97^
Ac_2-26_	FPR1/FPR2	Obesity-induced AF	♂ C57BL/6 mice HFD for 10 wk Ac_2-26_ 1 mg/kg i.p. for additional 10 wk	↓ AF frequency and duration dependent on CM AMPK activity, ↓ FBG, preserved insulin sensitivity, ↓ atrial diameter, ↓ atrial fibrosis, ↓ serum and atrial MDA, ↑ serum and atrial SOD, ↓ serum TG ↓ KCNH2 mRNA, ↑ SCN5A mRNA, ↑ CACNA1C and CACNA1D mRNA, ↑ PDK4 mRNA, ↑ CD36 mRNA ↓ p-RyR2, ↑ PPAR α, ↑ p-AMPK, ↑ FA oxidation, ↓ CM lipid droplet accumulation, ↓ CM apoptosis	Liu et al 2024^98^
Ac_2-26_	FPR1/FPR2	Myocarditis	♂ Sprague-Dawley rats CLP procedure Ac_2-26_ 1 mg/kg i.p 2 h before CLP	Preserved cardiac tissue integrity, ↓ cardiac cell apoptosis, preserved CM ultrastructure and sarcomere alignment	Zhang et al 2018^99^
CGEN-855A	FPR2	IR injury	♂ Albino mice LAD ligation (25 min) Reperfusion CGEN-855A 0.3 mg/kg,^*y*^ 1 mg/kg^*z*^ or 2 mg/kg^*aa*^ i.v. postreperfusion	↓ infarct AAR,^*z*,*aa*^↓ plasma cTnI^*z*,*aa*^	Hecht et al 2009^100^
CGEN-855A	FPR2	IR injury	♂ Sprague-Dawley rats LAD ligation (30 min) CGEN-855A 2 mg/kg i.v. 5 minutes before reperfusion Reperfusion (3 h)	↓ infarct AAR, ↓ cardiac PMN infiltration	Hecht et al 2009^100^
Act-389949	FPR2	Acute MI	♂ C57BL/6 mice Permanent LAD ligation Act-389949 1 mg/kg^*bb*^ or 10 mg/kg^*cc*^ by mouth daily for 3 days starting 24 h post-MI	↑ CD206^+^ cardiac macrophages^*bb*,*cc*^	Lupisella et al 2022^70^
Act-389949	FPR2	Acute MI	♂ Sprague-Dawley rats Permanent LAD ligation Act-3899491 mg/kg^*dd*^ or 10 mg/kg^*ee*^ by mouth daily for 6 wk starting 48 h post-MI	No preservation of EF or LV wall thickness^*dd*,*ee*^	Lupisella et al 2022^70^
Compound-17b	FPR1/FPR2	Acute MI	♂ C57BL/6 mice Permanent LAD ligation Compound-17b 50 mg/kg i.p. daily for 4 wk	Preserved cardiac function, ↓ infarct *Fpr1*, *Fpr2*, *CD68*, *Tnf-*α transcript levels	Qin et al 2017^68^
Compound-17b	FPR1/FPR2	IR injury	♂ C57BL/6 mice LAD ligation (40 min) Reperfusion (24 h^*ff*^ or 7 days^*gg*^) Compound-17b 50 mg/kg i.p. daily starting before reperfusion ♂ C57BL/6 mice LAD ligation (60 min) Reperfusion (48 h) Compound-17b 50 mg/kg i.p. daily starting before reperfusion	↓ infarct AAR,^*ff*^↓ infarct size,^*ff*^↓ plasma cTnI levels,^*ff*^↓ LV cell death,^*gg*^ no Δ LV collagen deposition,^*gg*^↓ LV weight^*gg*^ ↓ LV neutrophil infiltration, no Δ LV macrophage count, ↓ CM death, ↓ circulating neutrophils and lymphocytes, ↓ plasma IL-1 β	Qin et al 2017^68^
Compound-17b	FPR1/FPR2	Ang-II-induced HF	♂ C57BL/6 mice Ang-II 0.7 mg/kg s.c. daily for 28 days Compound-17b 50 mg/kg i.p. daily for 28 days	Preserved EF and FS, ↓ LV and CM hypertrophy, ↓ LV collagen deposition, preserved cardiac mitochondrial complexes I, II, and IV function	Singh et al 2024^101^

AAR, area at risk; Akt, protein kinase B; AMPK, AMP-activated protein kinase; CAC, circulating angiogenic cells; CACNA1C, α 1C subunit of L-type calcium channel Ca_v_1.2; CACNA1D, α 1D subunit of L-type calcium channel Ca_v_1.3; CD36, fatty acid translocase; CK, creatinine kinase; CLP, cecal ligation and puncture; cTnI, cardiac troponin I; EF, ejection fraction; FA, fatty acid; FBG, fasting blood glucose; FS, fractional shortening; HFD, high fat diet; KCNH2, α subunit of delayed rectifier current channel (I_Kr_); LA, left atria; LAD, left anterior descending coronary artery; LDH, lactate dehydrogenase; LVEDV, left ventricular end diastolic volume; LVDP, left ventricular diastolic pressure; LVSP, left ventricular systolic pressure; MAP, mean arterial pressure; MDA, malondialdehyde; MPO, myeloperoxidase; PDK4, pyruvate dehydrogenase kinase 4; PGF_2_α, prostaglandin F_2_α; PLB, phospholamban; PPAR α, peroxisome proliferator-activated receptor α; RPP, rate-pressure product; RyR2, ryanodine receptor 2; SCN5A; α subunit of sodium channel Na_v_1.5; SOD, superoxide dismutase; TG, triglycerides; TxB2, thromboxane B2 ; ♂, male; ♀, female; ↑, increase; ↓, decrease; Δ, change.

Superscript letters specify the effects associated with a particular dose of drug or treatment length in experimental designs with more than one drug dose or time point tested.

### A Myocardial infarction and ischemia-reperfusion repair

Most in vivo studies of SPM-GPCR agonism in heart disease have been in models of acute MI or IR injury. The studies primarily demonstrate improved left ventricular (LV) functional, structural, and hemodynamic parameters with exogenous SPM administration (Tables 1 and 2).^40^^,^^68^^,^^70^, ^71^, ^72^, ^73^, ^74^, ^75^, ^76^, ^77^, ^78^, ^79^^,^^92^, ^93^, ^94^, ^95^, ^96^, ^97^^,^^100^ Coordination of early and late inflammatory responses play a major role in MI and IR injury and subsequent recovery. Many immune cells recruited to the ischemic heart are derived from the spleen and bone marrow. After left anterior descending coronary artery occlusion, splenic-derived leukocytes are attracted to the LV.^76^ Data suggest that both the spleen itself and splenic-derived leukocytes release SPMs after MI, generating an SPM profile distinct from the LV.^76^ Sustained recruitment and impaired clearance of neutrophils exacerbates local tissue damage, whereas the delayed polarization of recruited monocyte-derived macrophages to a reparative phenotype prolongs removal of apoptotic neutrophils and necrotic tissue, perpetuating the local and systemic inflammatory state and promoting pathological remodeling.^102^ Exogenous SPM administration limits the chemotactic effects of circulating inflammatory cytokines.^73^^,^^74^^,^^78^ RvD1 administration post-MI hastens the clearance of neutrophils from the infarcted myocardium without affecting their early recruitment, which remains necessary for the initial inflammatory response responsible for eventual healing.^76^^,^^77^ BMS-986235 reduces the accumulation of cardiac neutrophils within the first 3 days of infarction, whereas RTP-026 reduces the levels of circulating activated neutrophils and monocytes before reaching the heart within 2 hours after reperfusion.^71^^,^^92^ Furthermore, SPMs and their analogs modulate macrophage phenotypic plasticity by driving them toward a more “reparative” phenotype, facilitating efferocytosis of necrotic cells and promoting repair at the infarct site.^40^^,^^70^^,^^71^^,^^77^^,^^93^ RTP-026 blunts classical monocyte recruitment to the rat heart with no effect on nonclassical monocyte subtypes.^92^ How exogenous administration of proresolution molecules post-MI coordinates communication between different organ systems and macrophage function needs further exploration.

SPMs also exert cardioprotective effects in MI and IR injury by preserving cardiac energetics and reducing oxidative stress. LxA4 improves postischemic mitochondrial ultrastructure and Na^+^/K^+^ ATPase activity,^73^^,^^74^ whereas LxA4 or RvD1 reduce malondialdehyde levels and improve superoxide dismutase activity in the heart.^74^^,^^78^ Ac_2-26_ is a peptidomimetic compound encompassing the active N-terminal amino acid sequence from endogenous annexin A1, and reduces cardiac neutrophil myeloperoxidase levels to blunt local oxidative stress.^96^

Considerations in MI and IR injury include both the timing of administration and the delivery method and formulation of SPMs. LxA4 administration before MI is more effective than post-MI treatment at preserving connexin 43 expression, correlating with reduced incidence of arrhythmias.^41^ However, translating these findings to humans will be challenging. Pretreatment of patients before acute MI is only possible if the drugs are safe enough to be administered chronically to at-risk individuals.^74^ In mice, RvE1 administered daily for the first 7 days after MI is protective, whereas waiting for 7 days before starting RvE1 impairs cardiac function and repair, suggesting that SPM modulation of the early inflammatory response post-MI is more effective than targeting the later reparative phase.^40^ As SPMs are large lipid molecules, the encapsulation of RvD1 into liposomes improves both molecular stability and efficiency of delivery to the infarcted myocardium, minimizing deposition in nontarget areas such as the liver.^77^^,^^79^

SPM administration in MI also exerts a positive feedback effect on SPM-GPCR receptor expression levels and SPM production in the heart and other organs such as the spleen, a rich reservoir for leukocytes.^77^^,^^79^ Compared with the characterization of SPM effects in isolated-heart models, investigation of SPM-GPCR signaling mechanisms in whole-animal models of heart disease is limited. However, one study shows that RvD1 enhances cardiac protein kinase B (Akt) activation, and that an Akt inhibitor abolishes the protective effects.^75^ These findings are also corroborated by an increase in cardiac phosphorylated-Akt with Ac_2-26_ administration.^97^ No studies have yet demonstrated GPCR-dependent lipid SPM effects in in vivo MI models with receptor knockout or SPM-GPCR selective antagonists. In the future, it will be of interest to confirm that the dramatic in vivo SPM effects are indeed GPCR-mediated and to decipher the detailed downstream pathways.

### B Myocarditis and septic cardiomyopathy

In murine septic cardiomyopathy induced by lipopolysaccharide (LPS) injection or cecal ligation and puncture, SPMs attenuate acute cardiac dysfunction, cytokine storm, and local cardiac damage.^85^^,^^87^^,^^89^^,^^90^ Ac_2-26_ signals through downstream PI3K/Akt to preserve cardiomyocyte and sarcomere ultrastructure, which rapidly erodes during acute systemic inflammation.^99^ A recently-identified and less characterized SPM, maresin-conjugate-in-tissue-regeneration 1 (MCTR), preserves mitochondrial function, enhances expression of mitochondrial biogenesis factors, maintains energy production in the acutely failing heart, and improves cardiac functional outcomes in response to LPS injection.^90^ As in MI, SPMs, particularly RvE1, demonstrate ROS scavenging properties by increasing cardiac glutathione levels and superoxide dismutase activity.^88^ Attenuation of cardiac hypertrophy and tumor necrosis factor α (TNF-α) levels in parasitic *Trypanosoma cruzi* infection are attributed to the synthesized version of AT-RvD1 and its effects at FPR2.^84^ FPR2 also plays a role in mediating cardioprotective effects in bacterial sepsis induced by cecal ligation and puncture.^103^

Activation and nuclear translocation of the transcription factor nuclear factor-κ B (NF-κ B) is responsible for upregulation of proinflammatory cytokines and components of nucleotide-binding and oligomerization domain-like receptor family pyrin domain-containing 3 inflammasome priming.^104^ Crosstalk exists between mitogen activated protein kinase (MAPK) signaling and NF-κ B transcriptional activity.^105^ Pretreatment with RvD1 or RvE1 attenuates LPS-induced p38 MAPK phosphorylation, NF-κ B activation, and proinflammatory cytokine gene upregulation in the mouse heart.^85^^,^^89^ RvE1 attenuates the activation of other MAPKs, including Jun N-terminal kinase and extracellular signal-regulated kinase (ERK)1/2 in the heart during acute inflammatory injury caused via cecal ligation and puncture.^87^ RvE1 also promotes cardiac activation of the PI3K/Akt pathway, which modulates metabolic processes such as autophagy and promotes cell survival.^87^^,^^88^ However, whether these changes are due to RvE1 binding to chemerin receptor 1 is unclear, and which cell type(s) in the heart are affected via these signaling networks is unknown.

Lastly, a recent study demonstrates benefit of the FPR2-selective small molecule, BMS-986235, and the dual FPR1/FPR2 agonist, Compound-43, in cardiac diastolic dysfunction induced by chronic inflammatory arthritis.^91^ FPR2 agonists modify the composition of cFB subpopulations (monocytic, structural, profibrotic, and inflammatory types). Furthermore, the authors distinguish between organ and cell type specific properties of FPR1 and FPR2. Both Compound-43 and BMS-986235 provide similar cardioprotection, likely driven through FPR2. The dual agonist, Compound-43, is ineffective for pulmonary protection, likely because of FPR1-driven activation of monocyte chemotaxis in synergy with the CCL2/CCR2-p44/42 MAPK axis.^91^

### C Atrial fibrillation

Research into SPM-GPCR signaling in models of AF remains rather limited. In a rat model of right-heart disease, RvD1 limits atrial electrical and structural remodeling, along with the promotion of AF.^83^ Similar effects have been noted in AF associated with LV dysfunction.^80^ Accumulating evidence suggests an important role for inflammatory mechanisms in AF pathogenesis, highlighting inflammation as a therapeutic target.^106^ Recent studies demonstrate that innate immune cells, including recruited and resident cardiac macrophages, play an important role in cardiac homeostasis and arrhythmias, including AF.^107^, ^108^, ^109^, ^110^ Ac_2-26_ has protective effects against AF associated with obesity, associated with both electrophysiological and metabolic changes.^98^ Additional work is needed to understand how and through which cell types SPMs act in AF.

### D Heart failure

Inflammation resolution also protects the heart from maladaptive remodeling in models of HF.^81^^,^^82^^,^^101^ In pressure overload-induced HF, RvD2 ameliorates cardiac dysfunction, CM hypertrophy and tissue fibrosis. RvD2 cardioprotective effects may stem from actions on immune cells, particularly F4/80^+^Ly6C^high^ recruited macrophages. RvD2 protection was abolished in Gpr18^−/−^ mice.^81^ Observational studies show that FPR2^−/−^ mice develop early-onset obesity-related diastolic dysfunction. Changes in cardiac ion-channel gene expression are evident as early as 4 months of age.^111^^,^^112^ Targeting of FPR2 with LxA4 attenuates LV fibrosis, CM apoptosis, and gene expression of proinflammatory cytokines in murine diabetes-induced diastolic dysfunction.^82^ Similar to Compound-43, Compound-17b also acts as a dual agonist at FPR1 and FPR2.^68^ Administration reduces cardiac remodeling and dysfunction after chronic angiotensin II (Ang-II) infusion in mice.^101^ Despite the FPR1/FPR2 biased action of Compound-17b and Compound-43, these 2 compounds may be distinct in their signaling profiles and breadth of cardioprotective actions.^68^

## IV Clinical correlation: Evidence for specialized proresolving mediator-G protein-coupled receptor involvement in human cardiac disease

In recent years, there have been an increasing number of studies assessing the systemic inflammatory profile of patients with cardiac disease and its contribution to disease development, progression, and prognosis. Limited observational studies suggest that the inflammation resolution process is disrupted in patients with heart disease, as reflected by alterations in SPM-GPCR expression and function, and a potential imbalance between SPM and proinflammatory lipid mediator synthesis (Table 3).^35^^,^^111^^,^^113^, ^114^, ^115^, ^116^, ^117^, ^118^, ^119^, ^120^, ^121^, ^122^, ^123^, ^124^

**Table 3:** Observational studies demonstrating potential involvement of SPMs or SPM-GPCRs in human cardiac disease

Disease State	Observation	Associated Outcome	References
Myocardial infarction/ischemia	↑ serum LxA4	↓ risk of MACE	Chen et al 2023^113^
	Early ↑ in PD1 and PD2 ↓ RvD5	STEMI	Fosshaug et al 2019^35^
	↓ plasma RvE1 in Black patients ↓ PD1 in White ♂ patients	STEMI	Halade et al 2020^114^
	↓ plasma RvD1 and MaR1	Coronary microvascular dysfunction	Keeley et al 2022^115^
	FPR2 gene enrichment in whole blood	Acute MI	Guo et al 2021^116^
	↑ FPR2 RNA in monocytes	Acute MI without plaque rupture	Qian et al 2022^117^
	↓ plasma RvD1	Acute coronary syndrome	Sun et al 2023^118^
	Cytoplasmic FPR2 localization in LV	Ischemia	Tourki et al 2020^111^
	FPR2 gene enrichment in ECs	Acute MI	Xiao et al 2022^119^
Heart failure	↓ plasma RvD1, impaired leukocyte production of RvD1 via 15-LOX, reduced leukocyte GPR32 expression	Presence of chronic HF with impaired T-cell responsiveness to RvD1	Chiurchi et al 2018^120^
	↑ LV and plasma GPR18 gene expression	Presence of chronic HF	Li et al 2020^121^
Septic cardiomyopathy	FPR2 gene enrichment	Presence of septic cardiomyopathy	Ma et al 2022^122^
Atrial Fibrillation	FPR2 gene enrichment in atrial appendage	Presence of persistent AF >3 months	Liu et al 2021^123^
	Atrial FPR2 gene enrichment	Presence of AF	Shi et al 2021^124^

MACE, major adverse cardiovascular event; STEMI, ST-segment elevation myocardial infarction; ↑, increase; ↓, decrease.

Studies of networks based on large patient datasets have identified FPR2 as a gene and protein hub, along with other inflammatory cytokines that is highly connected and enriched in acute MI, AF, and septic cardiomyopathy.^116^^,^^119^^,^^122^^,^^123^^,^^125^ In MI, FPR2 localizes to the cytosol of cells in the left ventricle and its gene expression is upregulated in circulating monocytes.^111^^,^^117^ Patients with HF have enhanced LV GPR18 gene expression and lower GPR32 expression in circulating leukocytes.^120^^,^^121^

Elevated levels of plasma LxA4 are associated with a lower risk of major adverse cardiovascular events after acute MI.^113^, ^114^, ^115^^,^^118^ MI patients with low levels of LxA4 and plasma high sensitivity C-reactive protein do not have a significantly lower risk of major adverse cardiovascular event compared with those with low LxA4 and high sensitivity C-reactive protein, who had the highest overall risk, suggesting that low levels of LxA4 are associated with adverse cardiovascular outcomes independent of the patient’s baseline systemic inflammatory status.^113^ Changes in circulating SPM levels may temporally reflect CVD during acute event progression.^35^ Plasma PD1 and PD2 levels rapidly double while RvD5 immediately declines after a ST-segment elevation myocardial infarction, even before troponin T levels reach their peak.^35^

Although studies in human populations are critical for assessing translational relevance, there are major limitations that need to be considered. The levels of SPMs and other lipid mediators are heavily influenced by diet and medications such as aspirin and statins.^126^ It is also difficult to determine whether any changes in SPMs and GPCRs are a cause or consequence of CVD pathogenesis or progression. Therefore, although these studies are useful hypothesis-generating tools, further validation in experimental models and clinical trials is essential.

## V Specialized proresolving mediator-G protein-coupled receptor pharmacology in heterologous overexpression systems

**Fig. 4:**
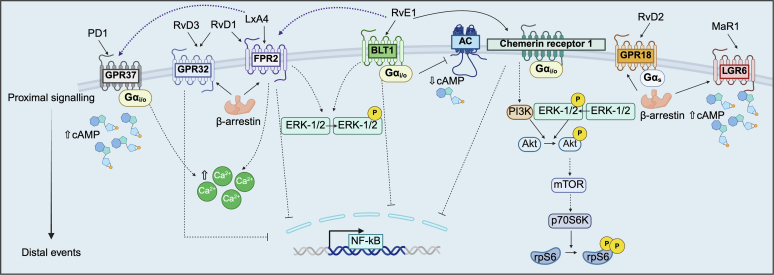
SPM-GPCR interactions induce a complex network of downstream signaling mechanisms in immortalized cell lines. Cells were transfected to induce heterologous overexpression of human SPM-GPCRs. Dashed blue lines indicate a weak level of evidence for the corresponding SPM-GPCR interaction. Dashed black dashed lines indicate distal signaling events where proximal effectors leading to the distal effects remain elusive. AC, adenylyl cyclase; LGR6, leucine-rich repeat-containing GPCR 6; mTOR, mammalian target of rapamycin.

**Fig. 5:**
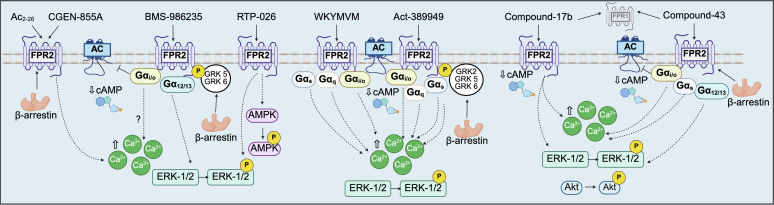
Synthetic peptide and molecule interactions with FPR2 signaling mechanisms in immortalized cell lines. Cells were transfected to induce heterologous overexpression of human FPR2. Dashed black lines indicate distal signaling events. Proximal effectors leading to the distal effects remain poorly defined. AMPK, AMP-activated protein kinase.

The use of immortalized cell lines for the overexpression of SPM-GPCRs allows researchers to interrogate the basic pharmacology of SPMs (Tables 4 and 5).^38^^,^^44^^,^^47^^,^^53^^,^^55^^,^^56^^,^^68^^,^^70^, ^71^, ^72^^,^^92^^,^^93^^,^^100^^,^^126^, ^127^, ^128^, ^129^, ^130^, ^131^, ^132^, ^133^, ^134^, ^135^, ^136^, ^137^, ^138^, ^139^, ^140^ Over the past 20 years, mechanistic studies have revealed a complex and intricate intracellular network downstream of SPM-GPCR activation (Figs. 4 and 5). However, findings are often conflicting, raising questions about the physiological implications of these results from heterologous overexpression systems.

**Table 4:** Effects of SPM administration in immortalized cell lines overexpressing human SPM-GPCRs

SPM; Receptor	Cell Line	Downstream Signaling and Effectors	Coupled G Protein	References
LxA4; FPR2	HEK 293	↑ [Ca^2+^]i	—	Merlin et al 2022^127^
LxA4; FPR2	CHO, HEK 293	Inactive; No Δ [Ca^2+^]i, cAMP, p-ERK1/2, β-arrestin recruitment	—	Hanson et al 2013^128^
LxA4; FPR2	HL-60	↑ p-ERK2	—	Christophe et al 2002^129^
LxA4; FPR2	RBL-2H3	↑ [Ca^2+^]i, No Δ p-ERK1/2	—	Bae et al 2003^130^
AT-LxA4; FPR2	CHO	Inactive; No Δ cAMP, GTP γ binding, [Ca^2+^]i	—	Planaguma et al 2013^131^
LxA4; GPR37	HEK 293	cAMP accumulation	—	Merlin et al 2022^127^
RvE1; FPR2	HEK 293	↑ [Ca^2+^]i	—	Merlin et al 2022^127^
LxA4, RvD1; GPR32	HEK 293	Inactive; No Δ cAMP	—	Merlin et al 2022^127^
RvD1, AT-RvD1; FPR2, GPR32	CHO, HEK 293	β-arrestin recruitment	—	Krishnamoorthy et al 2012^132^
RvD1; FPR2, GPR32	HeLa	β-arrestin recruitment ↓ TNF-α-stimulated NF-κ B gene activity	—	Krishnamoorthy et al 2010^55^
RvD3; GPR32	CHO	β-arrestin recruitment	—	Dalli et al 2013^53^
RvD1, RvD2, RvD4, RvD5, PD1, MaR1; EP4	HEK 293	↑ cAMP β-arrestin recruitment ↑ PGE_2_-induced cAMP	G α_s_ knockdown abolished effects G α_i/o_; PTX abolished SPM-potentiated PGE_2_ effects	Alnouri et al 2024^133^
RvE1; chemerin receptor 1	HEK 293	Inactive No Δ cAMP, GTP γ binding, β-arrestin recruitment, [Ca^2+^]i, ERK1/2	—	Merlin et al 2022^127^
RvE1; chemerin receptor 1	HEK 293	↓ TNF-α stimulated NF-κ B gene activity ↑ p-ERK1/2	G α_i/o_; PTX abolished effects	Arita et al 2005^38^
RvE1; chemerin receptor 1	CHO	↑ p-Akt, p-ERK1/2, p-rS6	—	Ohira et al 2010^44^
RvE1; BLT1	HEK 293	↓ cAMP ↑ p-ERK1/2, [Ca^2+^]i	—	Merlin et al 2022^127^
RvE1; BLT1	HeLa, HEK 293	↓ LTB_4_ stimulated NF-κ B gene activity, cAMP	G α_i/o_; PTX abolished effects	Arita et al 2007^47^
RvD2; GPR18	CHO	β-arrestin recruitment	G α_s_; CTX abolished binding impedance change measurements	Chiang et al 2015^56^
MaR1; LGR6	HEK 293	↑ cAMP β-arrestin recruitment	—	Chiang et al 2019^134^
PD1; GPR37	HEK 293	↑ in [Ca^2+^]i	G α_i/o_; PTX abolished effects	Bang et al 2018^135^

[Ca^2+^]i, intracellular calcium concentration; CHO, Chinese hamster ovary cells; CTX, cholera toxin; GTP, guanosine triphosphate; HL-60, human leukemia cells; LGR6, leucine-rich repeat-containing G protein-coupled receptor 6; RBL-2H3, rat basophilic leukemia cells; rS6, ribosomal protein S6; —, no information; ↑, increase; ↓, decrease; Δ, change.

**Table 5:** Effects of recombinant and synthetic SPM-GPCR agonist administration in immortalized cell lines overexpressing human SPM-GPCRs

Agonist; Receptor	Cell Line	Downstream Signaling and Effectors	Coupled G Protein	References
BMS-986235; FPR2	HEK 293, CHO	↑ in [Ca^2+^]i, ↓ cAMP, β-arrestin recruitment	—	Asahina et al 2020^72^
BMS-986235; FPR2	HEK 293, HL-60	G α_i_1, G α_i_2, G α_i_3, G α_o_A, G α_o_B, G α 12, Gα13 activation No Δ G α_q_, G α 11, Gα_s_ activity β-arrestin-1 and β-arrestin-2 recruitment ↑ phagocytosis, ↑ oxidative burst activity, chemotaxis	Direct G protein activation demonstrated via BRET biosensors	Garcia et al 2021^71^
BMS-986235; FPR2	CHO, HEK 293	↓ cAMP, G α_i_2 activation, β-arrestin-1 and β-arrestin-2 recruitment (GRK5 and 6-dependent)	Direct G protein activation demonstrated via BRET biosensors	Lupisella et al 2022^70^
BMS-986235; FPR2	HEK 293A	G α_i_2, G α_o_B, G α_s_ activation, β-arrestin-2 recruitment ↑ in [Ca^2+^]i, ↓ cAMP, ↑ p-ERK	Direct G protein activation demonstrated via BRET biosensors G α_i/o_; PTX abolished effects on [Ca^2+^]i	Peng et al 2024^136^
Compound-43; FPR1/FPR2	HEK 293	↑ in [Ca^2+^]i, p-ERK	—	Bena et al 2012^137^
Compound-43; FPR1/FPR2	HL-60	↑ in [Ca^2+^]i	—	Forsman et al 2011^138^
Compound-43; FPR1/FPR2	HEK 293, HL-60	G α_i_1, G α_i_2, G α_i_3, G α_o_A, G α_o_B, G α 12, Gα13 activation No Δ G α_q_, G α 11, Gα_s_ activity β-arrestin-1 and β-arrestin-2 recruitment ↑ oxidative burst acitivity, chemotaxis	Direct G protein activation demonstrated via BRET biosensors	Garcia et al 2019^93^
Compound-43; FPR1/FPR2	HEK 293	↓ cAMP, ↑ p-ERK, ↑ in [Ca^2+^]i, β-arrestin 2 recruitment	—	Merlin et al 2022^127^
Compound-43; FPR1/FPR2	HEK 293A	G α_i_2, G α_o_B, G α_s_ activation ↑ in [Ca^2+^]i, ↓ cAMP, ↑ p-ERK	Direct G protein activation demonstrated via BRET biosensors G α_i/o_; PTX abolished effects on [Ca^2+^]i	Peng et al 2024^136^
Compound-43; FPR1/FPR2	CHO	↑ p-ERK1/2, p-Akt (Ser474), p-Akt (Thr308), ↑ in [Ca^2+^]i, ↓ cAMP	—	Qin et al 2017^68^
RTP-026; FPR2	HEK 293	↑ p-ERK, p-AMPK	—	Chen et al 2023^92^
WKYMVM; FPR2	HL-60	β-arrestin recruitment	—	Forsman et al 2011^138^
WKYMVM; FPR2	HEK 293, CHO	↑ in [Ca^2+^]i, ↓ cAMP, ↑ p-ERK, β-arrestin recruitment	G α_i/o_; PTX abolished effects on cAMP	Hanson et al 2013^128^
WKYMVM; FPR2	HEK 293	↓ cAMP, ↑ p-ERK, ↑ in [Ca^2+^]i, β-arrestin 2 recruitment	—	Merlin et al 2022^127^
WKYMVM; FPR2	HEK 293A	G α_i_2, G α_o_B, G α_s_, G α_q_ activation, β-arrestin-1 and β-arrestin-2 recruitment ↑ in [Ca^2+^]i, ↓ cAMP, ↑ p-ERK FPR2 internalization and trafficking to early and late endosomes	Direct G protein activation demonstrated via BRET biosensors G α_i/o_; PTX abolished effects on [Ca^2+^]i	Peng et al 2024^136^
CGEN-855A; FPR2	CHO	↑ in [Ca^2+^]i	—	Hecht et al 2009^100^
Act-389949; FPR2	CHO	β-arrestin 2 recruitment	—	Lind et al 2019^139^
Act-389949; FPR2	CHO, HEK 293	↓ cAMP, G α_i_2 activation, β-arrestin-1 and β-arrestin-2 recruitment (GRK2, 5, and 6-dependent)	Direct G protein activation demonstrated via BRET biosensors	Lupisella et al 2022^70^
Act-389949; FPR2	HEK 293	↓ cAMP, ↑ in [Ca^2+^]i, β-arrestin 2 recruitment	—	Merlin et al 2022^126^
Act-389949; FPR2	HEK 293A	G α_i_2, G α_o_B, G α_s_, G α_q_ activation, β-arrestin-1 and β-arrestin-2 recruitment ↑ in [Ca^2+^]i, ↓ cAMP, ↑ p-ERK FPR2 internalization and trafficking to early and late endosomes	Direct G protein activation demonstrated via BRET biosensors G α_i/o_; PTX abolished effects on [Ca^2+^]i	Peng et al 2024^136^
Compound-17b; FPR1/FPR2	CHO	↑ p-ERK1/2, p-Akt (Ser474), p-Akt (Thr308), ↑ in [Ca^2+^]i, ↓ cAMP	—	Qin et al 2017^68^
Ac_2-26_	RBL-2H3, HEK 293	↑ in [Ca^2+^]i, β-arrestin-1 and β-arrestin-2 recruitment	—	Zhang et al 2020^140^

Akt, protein kinase B; AMPK, AMP-activated protein kinase; BRET, bioluminescence resonance energy transfer; [Ca^2+^]i, intracellular calcium concentration; CHO, Chinese hamster ovary cells; HL-60, human leukemia cells; RBL-2H3, rat basophilic leukemia cells; —, no information; ↑, increase; ↓, decrease.

### A Formyl peptide receptor 2

Conflicting results exist concerning LxA4 effects on intracellular calcium (Ca^2+^) levels in cells overexpressing FPR2. Some studies demonstrate the accumulation of Ca^2+^ in response to LxA4 administration,^127^^,^^130^ whereas others report no effect.^128^^,^^129^^,^^131^ Similar discrepancies exist regarding the activation of ERK1/2 by LxA4-FPR2 signaling.^128^, ^129^, ^130^ Although some studies suggest ERK1/2 phosphorylation increases in response to LxA4, others fail to show significant changes in ERK activation. These inconsistencies question the role of FPR2 as canonical GPCR responsible for mediating LxA4 effects. One study claims that LxA4 does not activate the heterologously expressed FPR2 receptor,^128^ whereas another suggests that RvE1 (rather than LxA4) binds and activates FPR2 to increase intracellular Ca^2+^,^127^ challenging the current dogma of SPM-GPCR pairs and forcing scientists to acknowledge that the pharmacology is not clear-cut. It is possible that heterogeneity among overexpression systems accounts for some of these conflicting results. Differences seen in immortalized cell lines regarding G protein bias, differing intracellular machinery responding to LxA4-FPR2 interactions, varying degrees of receptor overexpression, and use of different methods to quantify activation of downstream effectors might all contribute to the varying and sometimes conflicting results.

Signaling profiles of synthetic agonists at FPR2 have also been investigated. A common downstream effector of nearly all these agonists is increased intracellular Ca^2+^ levels with FPR2 activation.^68^^,^^72^^,^^100^^,^^127^^,^^128^^,^^136^, ^137^, ^138^^,^^140^ Pertussis toxin (PTX) pretreatment abolishes the effects of BMS-986235, Act-389949, WKYMVM, and Compound-43 on intracellular Ca^2+^, which suggests a possible G α_i/o_-activated, G βγ driven effect on Ca^2+^ rather than G α_q_-protein kinase C alone.^71^^,^^136^^,^^141^ Furthermore, Compound-43 was shown to be more biased toward Ca^2+^ mobilization compared with the other FPR1/FPR2 dual agonist, Compound-17b.^68^ This bias may contribute to the greater cardioprotective capacity of Compound-17b compared with Compound-43.^68^ Another study comparing 4 synthetic agonists, Compound-43, WKYMVM, Act-389949, and BMS-986235, shows that although all 4 ligands induce dissociation of G α_s_, G α_i2_, and G α_oB_ from G βγ and recruit β-arrestin 1 and 2, WKYMVM and Act-389949 are significantly more potent.^136^ This enhanced potency is also accompanied by increased trafficking of FPR2 away from the plasma membrane toward early and late endosomes in HEK 293 cells.^136^ Bias has also been demonstrated in agonist ability to recruit G protein receptor kinase (GRK) isoforms in conjunction with β-arrestin 1 and 2 recruitment.^70^ Whereas GRK 2, 5, and 6 promote β-arrestin recruitment with Act-389949, BMS-986235 is more selective toward GRK 5 and 6. Interaction of FPR2 with GRK 5 and 6 dampens the potency of Act-389949 on G α_i_ activation, whereas the response from BMS-986235 remains unaffected. It is evident that the complex pharmacology underlying synthetic agonist actions at FPR2 warrants further investigation in physiologically relevant cell types such as immune cells.

### B G protein-coupled receptor 32 and 18

GPR32 is a pseudogene in mice and rats, making its investigation in these in vivo models difficult.^142^ Heterologous overexpression systems are therefore a useful tool for understanding the pharmacology of GPR32. In these cell systems, RvD1 potently promotes β-arrestin recruitment with an EC_50_ of approximately 8.8 × 10^−12^ M,^55^^,^^132^ whereas LxA4 has no effect on cAMP levels.^127^ Likewise, RvD2 activation of GPR18 also increases β-arrestin recruitment.^56^ GPR18 possesses high affinity and specificity for RvD2 with a *K*_*d*_ of under 10 nM.^56^ This interaction is sensitive to cholera toxin, which abolishes GPR18-RvD2 binding impedance measurements, suggesting that RvD2-GPR18 may be coupled to G α_s_, at least in Chinese hamster ovary cells.^56^ Phosphodiesterase-4 inhibitors,^143^ which prevent the breakdown of cAMP, have known anti-inflammatory effects.^144^ Given that some SPMs, such as RvD2, are coupled through GPR18 to G α_s_, it would be of interest to determine whether coadministration of RvD2 and phosphodiesterase-4 inhibitors mediate synergistic anti-inflammatory effects by increasing and sustaining levels of intracellular cAMP.

### C Chemerin receptor 1 and leukotriene B_4_ receptor

Binding to chemerin receptor 1 in HEK 293 cells is specific to RvE1 and not its metabolic precursors, eicosapentaenoic acid or 18R-hydroxyeicosapentaenoic acid (HEPE), with a *K*_*d*_ of approximately 11 nM.^38^ RvE1 potently inhibits NF-κ B activation through chemerin receptor 1 in transfected HEK 293 cells with an EC_50_ of 1 nM, an effect that is abolished with PTX pretreatment, suggesting a G α_i/o_ coupling mechanism.^38^ Additionally, RvE1-chemerin receptor 1 signaling promotes ERK1/2 phosphorylation and activation.^38^^,^^44^ Conventionally, activation of NF-κ B activity and chemokine signaling is thought to be MAPK-activation dependent.^145^ Furthermore, RvE1 reduces ERK1/2 activity in the heart, coinciding with an attenuated inflammatory response.^87^ Therefore, ERK1/2 activation may be an artifact of the overexpression model, or, more interestingly, may serve an unknown role in mediating the protective effects of RvE1. Similarly, BLT1 also transduces signals from RvE1 similar to those mediated by chemerin receptor 1, including PTX-sensitive inhibition of NF-κ B activity and cAMP reduction, as well as ERK1/2 activation.^47^^,^^127^ The overlapping downstream receptor activation and NF-κ B inhibition may indicate some form of functional redundancy.

### D Other specialized proresolving mediator-G protein-coupled receptors

Binding of MaR1 to its receptor, leucine-rich repeat-containing GPCR 6, increases cAMP and promotes β-arrestin recruitment to the receptor.^134^ Binding of PD1 to GPR37 increases intracellular Ca^2+^ concentration; this effect is abolished by PTX treatment, suggesting G α_i/o_-coupling.^135^ LxA4 may also serve as a ligand at GPR37, conventionally known as the receptor for PD1.^127^ In HEK 293 cells overexpressing human GPR37 and treated with a series of SPMs, only LxA4 affects cAMP accumulation.^127^ The direct recruitment of G α_s,_ is further validation of this ligand-receptor pair; effects in primary cell lines have yet to be demonstrated. D-series resolvins, protectins, and maresins act as positive allosteric modulators at the prostaglandin E_2_ (PGE_2_) receptor, EP4.^133^ Nanomolar concentrations of these compounds enhance PGE_2_-induced cAMP formation through G α_s_, but also increase G α_i/o_ recruitment.

### E Moving beyond heterologous overexpression systems

These heterologous overexpression studies serve an essential purpose in understanding the basic pharmacology of SPM-GPCRs. These findings can also be applied to some physiological situations in models of cardiac disease. However, direct assessment of these mechanisms in models of cardiac disease remains to be accomplished. The conclusions that can be drawn about signaling patterns assessed in heterologous overexpression systems cannot be directly extrapolated to more physiologically relevant cell types, such as cardiomyocytes, primary monocytes, and tissue macrophages. These receptors may require heterodimerization or conformational changes that are not possible in a synthetic overexpression system.^48^^,^^146^ Endogenous expression patterns and localization of SPM-GPCRs may also differ significantly in primary cells compared with immortalized cell lines with GPCR overexpression.^147^ However, SPM-GPCR signaling networks are both cell-type and disease-context dependent.^146^ Transition to more physiological cell systems to investigate SPM-GPCR signaling has begun, and will be critical in understanding the therapeutic usefulness of SPMs in heart disease. We discuss below what is known about SPM-GPCR signaling in various cell types known to be important in cardiac function and disease.

## VI Specialized proresolving mediator-G protein-coupled receptor actions on immune cells

**Fig. 6:**
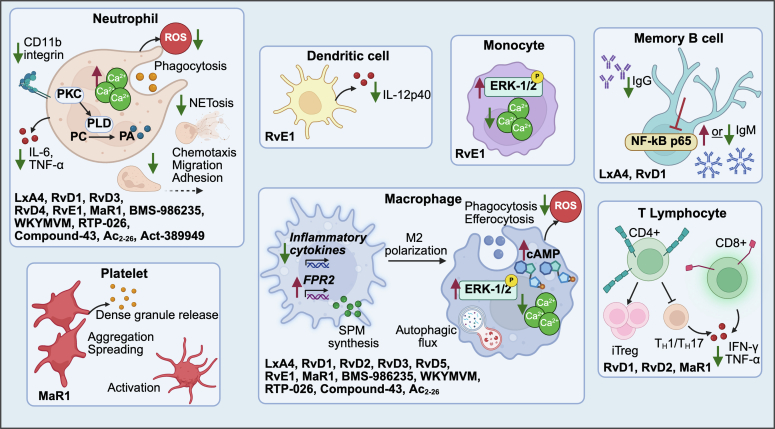
SPM-GPCR signaling induces changes in cells of the immune system. Primary immune cells when stimulated to induce activation, show subsequent effects when treated with SPMs. PA, phosphatidic acid; PC, phosphatidylcholine; PKC, protein kinase C; PLD, phospholipase D.

Immune cells play important roles in heart disease and in normal cardiac function.^1^, ^2^, ^3^^,^^107^^,^^108^ Some of the best-documented effects of SPMs have been identified by investigations of immune cells in culture and in vivo (Fig. 6; Tables 6 and 7).^38^^,^^40^^,^^44^, ^45^, ^46^, ^47^^,^^53^^,^^55^^,^^69^^,^^71^^,^^72^^,^^79^^,^^81^^,^^87^^,^^91^, ^92^, ^93^^,^^126^^,^^128^^,^^130^^,^^131^^,^^133^, ^134^, ^135^^,^^137^, ^138^, ^139^^,^^143^^,^^148^, ^149^, ^150^, ^151^, ^152^, ^153^, ^154^, ^155^, ^156^, ^157^, ^158^, ^159^, ^160^, ^161^, ^162^, ^163^, ^164^, ^165^, ^166^, ^167^, ^168^, ^169^, ^170^, ^171^, ^172^, ^173^ Results from primary immune cells in vitro are vital for understanding SPM-GPCR signaling in a more physiologically relevant context.

**Table 6:** Effects of SPM treatment in vitro on primary immune cells

Immune Cell Type	SPM Agonist	Model	Downstream Signaling and Effectors	Evidence of Receptor-Dependency *or* Coupled G Protein	References
Macrophage/monocyte	LxA4	PBMC-derived macrophages from patients with atherosclerosis	↓ oxLDL uptake, foam cell formation	FPR2-dependent; effects blocked by Boc-2 antagonist	Mai et al 2018^148^
	AT-LxA4	LPS and IFN-γ stimulated murine peritoneal macrophages	↑*Fpr2* gene expression	—	Kain et al 2017^149^
	AT-LxA4	Murine peritoneal macrophages, human PBMCs	↑ p-Akt, p-ERK2, ERK2-LC3 interaction, p62 degradation, LC3-I to LC3-II processing, autophagosome and autolysosome formation, autophagic flux ↓ p-CAMKII, [Ca^2+^]i	—	Prieto et al 2015^150^
	RvD1	Elastase-treated murine splenic macrophages	↓ ROS, MDA, GSH, NOX2 p-p47^*phox*^, Nrf2 nuclear translocation, HMGB1 secretion	FPR2-dependent; effects abolished in macrophages from FPR2^(−/−)^ mice	Filiberto et al 2022^151^
	RvD1	AA or LTA_4_ stimulated murine BMDM	↓ p-p38, p-MK2, p-CAMKII, p-5-LOX, [Ca^2+^]i ↑ LxA4 synthesis Cytosolic 5-LOX localization	—	Fredman et al 2014^152^
	RvD1	Murine BMDM cocultured with NCs	↓ RhoA-p-MLC signaling ↑ CDC42 activation, cytoskeletal rearrangement promoting calreticulin release, NC whole-cell engulfment	—	Gerlach et al 2020^153^
	RvD1	Human PBMC-derived macrophages exposed to zymosan A or apoptotic PMNs	↑ phagocytic activity	FPR2 and GPR32-dependent; receptor overexpression enhanced effects, siRNA knockdown abolished effects	Krishnamoorthy et al 2010^55^
	RvD1	Murine BMDM, murine gastrocnemius F4/80^+^ macrophages after HLI	Enrichment of vascular development and regulation and immunity genes	—	Sansbury et al 2020^154^
	RvD1	LPS and IFN-γ stimulated murine BMDM	↑ apoptotic CM efferocytosis, SPM production, hypoxic HUVEC migration and angiogenesis	—	Weng et al 2022^79^
	RvD1, RvD5	Human PBMC-derived macrophages exposed to *Escherichia coli*	↑ phagocytosis ↓ proinflammatory gene expression	GPR32-dependent; receptor overexpression enhanced effects	Chiang et al 2012^155^
	RvD2	Human PBMC-derived macrophages exposed to *E. coli* or apoptotic PMNs	↑ cAMP, phagocytosis, efferocytosis	GPR18-dependent; receptor overexpression enhanced effects, shRNA knockdown abolished effects	Chiang et al 2015^135^
	RvD2	Peritoneal macrophages from Ang-II-infused mice	M2 polarization	—	Diaz Del Campo et al 2023^156^
	RvD2	Murine BMDM LPS and IFN-γ stimulation	↓ TNF-α, IL-1 β, IL-6 mRNA ↓ CD80, CD86, iNOS, Ly6C^*high*^ macrophages ↓ p-p65 and p-STAT1	GPR18-dependent; effects abolished in BMDM isolated from GPR18^(−/−)^ mice	Zheng et al 2024^81^
	RvD3, AT-RvD3	Murine peritoneal macrophages ad human PBMC-derived macrophages incubated with zymosan or apoptotic PMNs	↑ phagocytosis and efferocytosis	GPR32-dependent; receptor overexpression enhanced effects	Dalli et al 2013^53^
	RvD5	LPS-treated human PBMC-derived macrophages incubated with zymosan or apoptotic PMNs	↑ PLD activity, p-S6P, S6K activity, M2 macrophage polarization, efferocytosis	—	Ganesan et al 2020^157^
	RvD1, RvD2, RvD5, PD1, MaR1, MaR2	Murine BMDM, murine BMDM stimulated with LPS	↑ PGE_2_-induced cAMP G α_i/o_ activation ↓ TNF-α ↑ G α_i/o_ -dependent phagocytosis activity	EP4-dependent; effects abolished in BMDMs from mice with macrophage-specific knockdown of EP4	Alnouri et al 2024^133^
	RvE1	LPS-stimulated murine BMDM	↑ phagocytosis ↓ proinflammatory gene expression	—	Chen et al 2020^87^
	RvE1	LPS or IL-4-treated mouse peritoneal macrophages	↓ transmigration	Chemerin receptor 1-dependent; effects reduced with siRNA	Lui et al 2018^40^
	RvE1	Human PBMC-derived macrophages	↑ rS6 phosphorylation via Akt-mTOR pathway ↑ phagocytosis of zymosan	Chemerin receptor 1-dependent; effects blocked by chemerin receptor 1 neutralizing Ab	Ohira et al 2010^44^
	RvE1	Human PBMC	↑ p-ERK1/2	—	Arita et al 2005^38^
	RvE1	LTB_4_-stimulated human PBMC	↓ [Ca^2+^]i	—	Arita et al 2007^47^
	MaR1	Murine peritoneal macrophages, human PBMC-derived macrophages incubated with *E. coli*	↑ phagocytosis, cAMP, p-ERK1/2, p-CREB	LGR6-dependent; receptor overexpression enhanced effects, siRNA knockdown abolished effects	Chiang et al 2019^134^
	MCTR	Human PBMC-derived macrophages incubated with *E. coli*	↑ phagocytosis	CysLT1-dependent; MK571 receptor antagonist abolished effect	Chiang et al 2018^158^
	MaR1 + RvD2	TNF-α and IFN-γ treated mouse peritoneal macrophages	↓ TNF-α, IL-6 secretion ↑ TGF-β release	—	Viola et al 2016^159^
Neutrophil	LxA4	Human PMNs	↑ [Ca^2+^]i, PKC-induced PLD activity, PA generation ↑ chemotaxis	G α i/o; PTX treatment abolished effects	Bae et al 2003^130^
	LxA4	Human PMNs	No Δ [Ca^2+^]i transient	—	Hanson et al 2013^128^
	LxA4	Human PMNs from patients with atherosclerosis incubated with *E. coli*	↓ ROS, oxidative burst, CD11b integrin expression	—	Kraft et al 2022^126^
	AT-LxA4	Human PMNs stimulated with IL-8	↓ chemotaxis, migration No Δ viability	—	Planaguma et al 2013^131^
	RvD1	Human PMNs	↓ actin polymerization, CD11b integrin expression No Δ cAMP or [Ca^2+^]i	G α i/o; PTX treatment abolished effects	Krishnamoorthy et al 2010^55^
	RvD1	Human PMNs exposed to inflamed venule shear flow rate	↓ interaction with endothelial cells ↑ zymosan phagocytosis	GPR32 and FPR2-dependent; effects abolished with receptor neutralizing Ab	Norling et al 2012^160^
	RvD3, AT-RvD3	Human PMNs exposed to TNF-α treated HUVECs	↓ migration through endothelial cell layer	—	Dalli et al 2013^53^
	RvD4	PMNs from blood of RvD4-treated mice	↓ ionomycin-induced NET formation	—	Cherpokova et al 2019^161^
	RvE1	Human PMNs	↓ migration across HUVEC monolayer	BLT1-dependent; effects reduced by siRNA and receptor antagonist U-75302	Liu et al 2018^46^
	RvE1	Human PMNs	↓ LL-37-induced LTB_4_ release	—	Wan et al 2011^162^
Dendritic cell	RvE1	Murine splenic CD11c^+^ DC exposed to *Toxoplasma gondii* pathogen	↓ IL-12p40 production	Chemerin receptor 1-dependent; effect abolished with siRNA	Arita et al 2005^38^
	RvE1	Murine splenic CD11c^+^ DC treated with Pam_3_CSK_4_	↓ DC maturation ↓ IL-2 and IL-6	—	Oner et al 2021^163^
T lymphocyte	RvD1, RvD2, MaR1	Human PBMC-derived T lymphocytes activated with PMA/ionomycin	↓ cytokine production, CD4 T-cell differentiation to T_H_ cells ↑ iTreg generation	FPR2 and GPR32-dependent; RvD1 effects abolished by receptor neutralizing Ab	Chiurchiu et al 2016^143^
	RvD1	Human PBMC-derived T lymphocytes activated with anti-CD3/anti-CD28	↑ iTreg generation ↑ TGF-β and IL-10 secretion ↓ T_H_ 17 differentiation ↓ IL-17 secretion	—	Cheng et al 2021^164^
	RvD1	Spleen-derived murine naïve CD4^+^ T cells activated with anti-CD3/anti-CD28	↓ T_H_ differentiation	—	Huang et al 2024^165^
	RvD1	Liver-derived murine naïve T cells activated with anti-CD3/anti-CD28	↓ IFN-γ production ↓ Kupffer cell activation with conditioned media	—	Navarro-Corcuera et al 2025^166^
	RvD5	Spleen-derived murine naïve CD4^+^ T cells	↑ iTreg generation ↓ T_H_ 17 differentiation	—	Yamada et al 2021^167^
	RvE1	Spleen-derived murine naïve CD4^+^ T cells activated TGF-β/IL-6/anti-CD28 or DC coculture	↓ T_H_ 17 differentiation ↓ IL-17, IL-2, and CCL20 secretion ↓ CCR6 and CD25 expression	—	Oner et al 2021^163^
Memory B cell	LxA4	Activated human B cells	↓ NF-κ B p65 nuclear translocation, IgM and IgG production, proliferation	FPR2-dependent; effect blocked by receptor antagonist Boc-2	Ramon et al 2014^168^
	RvD1	Activated human B cells	↑ IgM production	—	Ramon et al 2012^169^
Platelet	RvD1, MaR1	Human platelets activated with ADP and thrombin	↑ aggregation, spreading, hemostatic activation, dense granule release ↓ inflammatory mediator release	—	Lannan et al 2017^170^
	RvE1	Human platelet-rich plasma activated with ADP	↓ P-selectin surface expression, actin polymerization, aggregation No Δ [Ca^2+^]i	Chemerin receptor 1-dependent; overexpression enhanced effects	Fredman et al 2010^45^

Ab, antibody; BMDM, bone marrow-derived macrophage; [Ca^2+^]i, intracellular calcium concentration; CAMKII, calcium-calmodulin-dependent protein kinase II; Cdc42, Cdc42 Rho-type GTPase; CREB, cAMP response element-binding protein; DC, dendritic cell; GSH; glutathione; HLI, hind limb ischemia; HMGB1, high mobility group box protein 1; HUVEC, human umbilical vein endothelial cell; IFN-γ, interferon gamma; iTreg, induced T regulatory cell; LC3, microtubule-associated protein 1A/1B-light chain 3; LGR6, leucine-rich repeat-containing G protein-coupled receptor 6; LTA_4_, leukotriene A_4_; MDA, malondialdehyde; MLC, myosin light chain; mTOR; mammalian target of rapamycin; NC, necroptotic cell; NET, neutrophil extracellular trap; NOX2 p47phox, NADPH oxidase 2 p47phox; Nrf2; nuclear factor erythroid 2-related factor 2; oxLDL, oxidized low density lipoprotein; PA, phosphatidic acid; PBMC, peripheral blood mononuclear cell; PKC, protein kinase C; PLD, phospholipase D; PMA, phorbol myristate acetate; RhoA, Rho GTPase; rS6, ribosomal protein 6; S6K, ribosomal protein S6 kinase *β*1; S6P, ribosomal protein 6; shRNA, short hairpin RNA; siRNA, short interfering RNA; —, no information; ↑, increase; ↓, decrease; Δ, change.

**Table 7:** Effects of synthetic SPM-GPCR agonist treatment in vitro on primary immune cells

Agonist; Receptor	Immune Cell Type	Downstream Signaling and Effectors	Evidence of Receptor-Dependency *or* Coupled G Protein	References
BMS-989235; FPR2	Mouse peritoneal macrophages	↑ phagocytosis	—	Asahina et al 2020^72^
BMS-986235; FPR2	Mouse peritoneal macrophages, Human PMNs	↑ phagocytosis ↑ IL-10 mRNA, ↑ MCP-1 mRNA Induction of apoptosis	Abolished in macrophages from FPR2^−/−^ mice	Garcia et al 2021^71^
WKYMVM; FPR2	Human PMNs	↑ [Ca^2+^]i, PKC-induced PLD activity, PA generation ↑ chemotaxis, ↑ superoxide generation, ↑ cytosolic PLA_2_ activation and AA release, ↑ p-ERK1/2, p-JNK	G α i/o; PTX treatment abolished effects on Ca^2+^	Bae et al 2003^130^
WKYMVM; FPR2	Mouse BMDMs	↑ p-ERK	Abolished in FPR2^−/−^ cells	Dufton et al 2010^171^
WKYMVM; FPR2	Mouse peritoneal neutrophils	↑ ROS release	Partially reduced with pre-desensitization of receptor by Compound-43	Forsman et al 2011^138^
WKYMVM; FPR2	Human PMNs	↑ [Ca^2+^]i	—	Hanson et al 2013^128^
Compound-43; FPR1/FPR2	Human PMNs	↑ [Ca^2+^]i	—	Bena et al 2012^137^
Compound-43; FPR1/FPR2	Mouse BMDMs	↑ p-ERK	Abolished in FPR2^−/−^ cells	Dufton et al 2010^171^
Compound-43; FPR1/FPR2	Mouse peritoneal neutrophils	↑ ROS release	Partially reduced with pre-desensitization of receptor by WKYMVM	Forsman et al 2011^138^
Compound-43; FPR1/FPR2	Mouse peritoneal macrophages	↑ phagocytosis, ↑ IL-10 mRNA, ↓ IL-6 mRNA	Abolished in macrophages from FPR2^−/−^ mice	Garcia et al 2019^93^
Compound-43; FPR1/FPR2	Human PBMCs	Potentiated CCL2-induced chemotaxis ↑ p-44/42 MAPK	Chemotaxis attenuated with FPR2 antagonist, WRW4	Margraf et al 2025^91^
Compound-43; FPR1/FPR2	Human PMNs	↓ chemotaxis and [Ca^2+^]i response to IL-8, C5a, or LTB_4_ ↓ CXCR1, CXCR2, C5a receptor, and BLT1 surface expression, ↑ CD11b surface expression	—	Sogawa et al 2011^172^
Compound-43; FPR1/FPR2	Mouse BMDNs and PMNs	↓ chemotaxis and [Ca^2+^]i response to C5a, LTB_4_, or KC ↓ CXCR2 surface expression	—	Sogawa et al 2011^69^
RTP-026; FPR2	Human PMNs Human PBMCs	Induction of apoptosis, ↑ phagocytosis ↑ phagocytosis	— —	Chen et al 2023^92^
Ac_2-26_; FPR1/FPR2	Mouse PMNs and PBMCs, Human PMNs and PBMCs	↓ VCAM-1 and ICAM-1 binding to surface β_1_ and β_2_ integrins in response to chemokines ↓ GTP-bound Rap1 in response to CCL5 ↓ adhesion to TNF-α-activated HUVECs	—	Drechsler et al 2015^173^
Ac_2-26_; FPR1/FPR2	Mouse BMDMs	↑ p-ERK, no effect on migration	Abolished in FPR2^−/−^ cells	Dufton et al 2010^171^
Act-389949; FPR2	Human PMNs	↑ [Ca^2+^]i, ROS production, chemotaxis, degranulation, ↑ surface CD11b expression, cytoskeletal actin-dependent NADPH-oxidase activation	[Ca^2+^]i and ROS effects independent of G α_q_ activation	Lind et al 2019^139^

BMDM, bone marrow-derived macrophages; BMDN, bone marrow-derived neutrophils; [Ca^2+^]i, intracellular calcium concentration; CCL5, chemokine C-C motif ligand 5; GTP, guanosine triphosphate; HUVEC, human umbilical vascular endothelial cell; KC, keratinocyte-derived cytokine; JNK, Jun N-terminal kinase; MCP-1, monocyte chemoattractant protein 1; PA, phosphatidic acid; PBMC, peripheral blood mononuclear cell; PKC, protein kinase C; PLA_2_; phospholipase A_2_; PLD, phospholipase D; —, no information; ↑, increase; ↓, decrease.

### A Macrophages and monocytes

Macrophages and monocytes abundantly express SPM-GPCRs, making them prime cellular candidates for SPM actions.^38^^,^^56^^,^^135^ Very little work has been done on SPM actions in macrophages isolated from the heart, so the abundance of information available about SPM effects on macrophages (summarized as briefly as possible here) comes mostly from noncardiac sources. SPMs promote plasticity of macrophages toward a more reparative phenotype, possibly via resolvin-enhanced phospholipase D activity.^156^^,^^157^ Reparative macrophage characteristics include enhanced efferocytotic capability for apoptotic cells and phagocytic activity, as well as reduced inflammatory gene expression and cytokine release.^44^^,^^53^^,^^55^^,^^56^^,^^79^^,^^87^^,^^134^^,^^153^^,^^155^^,^^158^ BMS-986235 and Compound-43 increase IL-10 mRNA levels, phagocytosis, and chemotaxis, possibly driven by activation of p44/42 MAPK. This is abolished in macrophages from FPR2 deficient mice.^71^^,^^72^^,^^91^^,^^93^^,^^171^ PD1 also causes macrophages to adopt a proregenerative phenotype.^154^^,^^174^ Through these functions, resolution of inflammation is actively initiated.^175^^,^^176^ A positive feedback loop is activated, enhancing SPM-GPCR expression on macrophages and promoting further SPM synthesis and release.^79^^,^^149^^,^^152^ RvD1 treatment favors localization of 5-LOX in the cytosol, enhancing LxA4 production,^152^ with LxA4 acting in a paracrine and autocrine manner to sustain the proresolution macrophage phenotype.

Common downstream signaling networks include activation of the PI3K/Akt signaling pathway and MAPKs such as ERK1/2, along with downstream phosphorylation of the cyclic AMP response-element binding protein.^134^ RvD5 acts as an allosteric modulator at the PGE_2_ receptor, EP4, inhibiting the suppression of PI3K/Akt by PGE_2_ and increasing PI3K-dependent phagocytic activity in murine bone marrow-derived macrophages.^133^ RvE1-induced p-ERK1/2 activation in monocytes is G α_i/o_ coupled, which supports previous studies showing PTX-induced abolition of p-ERK1/2 increase in RvE1-treated HEK 293 cells.^38^ Findings in immortalized cell lines and primary immune cell studies complement each other by showing that MaR1 induces a cAMP increase in HEK 293 cells overexpressing leucine-rich repeat-containing GPCR 6, as well as in native human and mouse macrophages.^134^

Effects of SPMs go beyond direct inflammation resolution and macrophage polarization. RvD1 and the aspirin-triggered epimeric form of LxA4, AT-LxA4, promote autophagic flux in murine macrophages and human blood-derived monocytes.^150^ Incubation of these cells with LxA4 promotes the formation of autophagosomes and their fusion with lysosomes in a mammalian target of rapamycin-independent manner.^108^ Accordingly, SPMs play a role in regulating macrophage lifespan and homeostasis, which may indirectly promote the resolution of inflammation.

In the context of CVD, peritoneal macrophages isolated from mice treated with Ang-II and RvD2 have greater expression of genes associated with reparative/proresolution phenotypes compared with Ang-II treatment alone.^156^ When conditioned media from macrophages treated with TNF-α in the presence of MaR1 and RvD2 is put on aortic VSMCs, this results in greater collagen expression by VSMCs that enhances the stability of fibrous caps in atherosclerotic lesions, reducing the risk of rupture.^159^ When the transforming growth factor-β (TGF-β) receptor was blocked on VSMCs exposed to SPM-treated macrophage media, the beneficial effects of enhanced VSMC collagen production were attenuated, suggesting an important role for SPM-induced macrophage TGF-β release on VSMC stabilization of atherosclerotic plaques.^159^ VSMCs treated with the conditioned media from RvD1 and elastase cotreated macrophages produce less ROS and have lower levels of matrix metalloproteinase-2 activity, which preserves the structural integrity of vascular tissue, reducing the risk of aneurysm formation and ruptures.^151^ Similarly, RvD1-treated murine bone marrow-derived macrophages incubated with ECs cultured under hypoxic conditions promote EC migration and proangiogenic phenotypes.^79^ However, SPMs influence the release of mediators from macrophages that exert effects on other cell types in their microenvironment.

Although steps have been taken to investigate the effects of SPMs on macrophages and monocytes in CVD, *how* SPM signaling on macrophages and monocytes leads to cardiac phenotypic changes and effects via their GPCRs is still unclear, as is the nature of crosstalk with CMs, fibroblasts, VSMCs, and ECs.

### B Neutrophils

Neutrophils are the first-responders to sites of inflammatory injury and tissue damage and are essential players in the transcellular biosynthesis of SPMs.^32^^,^^33^ Although they are essential for acute protection of the host, persistent neutrophil activity is a major cause of unresolved and chronic inflammation leading to disease, including CVD.^102^ SPM treatment attenuates neutrophil migration and chemotaxis and reduces the expression of surface integrins, which aid adhesion to ECs and promote neutrophil infiltration into tissues.^46^^,^^53^^,^^55^^,^^126^^,^^131^ Binding of surface β_1_ and β_2_ integrins with intercellular adhesion molecule-1 (ICAM-1) and vascular cell adhesion molecule-1 (VCAM-1) in response to CCL2, CCL5, and CXCL1 chemokines is lowered with Ac_2-26_ treatment, which interferes with integrins to adopt active conformations.^173^ Consequently, Ac_2-26_-treated neutrophils have reduced adhesion when perfused over TNF-α-primed human umbilical vascular ECs.^173^ Compound-43 blunts the capacity of neutrophils to respond to CXC chemokines by reducing CXCR1 and CXCR2 surface expression levels.^69^^,^^172^

Neutrophils express SPM receptors, including FPR2.^128^ However, dual agonists such as Compound-43 may signal preferentially through FPR1 (rather than FPR2) on neutrophils to increase intracellular Ca^2+^ levels and superoxide anion production.^138^ There is also uncertainty about whether SPMs such as LxA4 signal through FPR2 in neutrophils. One study demonstrated that LxA4 increases neutrophil intracellular Ca^2+^ levels in a PTX-sensitive manner, suggesting FPR2 coupling to G α_i/o_.^130^ Other studies, also using human PMNs, concluded that there are no FPR2-dependent effects of LxA4 alone, but rather that LxA4 may function as an allosteric modulator in the presence of other ligands bound to the orthosteric binding site.^128^^,^^131^ Furthermore, LxA4 may function as an inverse agonist at the neutrophil FPR2 receptor. The antimicrobial peptide LL-37 binds to neutrophil FPR2 to stimulate inflammatory LTB_4_ release.^162^ LxA4 alone is unable to affect LTB_4_ release from neutrophils but inhibits LTB_4_ release in the presence of LL-37. Thus, mediation of SPM effects on neutrophils may be more complicated than a simple agonist-receptor relationship and further research is needed.

PMNs isolated from patients with atherosclerosis exhibit a hyperreactive phenotype and a unique response to LxA4 compared with those from healthy patients.^126^ LxA4 reduces oxidative burst in atherosclerotic-derived PMNs challenged with *Escherichia coli*, whereas LxA4 increases ROS production from healthy PMNs.^126^ It appears that effects of SPM treatment may be more gradual than an “on-off” switch, with different internal activation states of PMNs modulating the biological effect of LxA4. If so, SPMs could be used to fine-tune the neutrophil response in diseases with varying levels of inflammatory burden.

### C Other cells of the innate and adaptive immune systems

A handful of work has been done to investigate SPM-GPCR signaling in dendritic cells, platelets, and cells involved in the adaptive immune response (Fig. 6; Tables 6 and 7). Splenic dendritic cells express chemerin receptor 1 mRNA, and RvE1 reduction of IL-12 secretion is chemerin receptor 1-dependent.^38^ CD4^+^ T cells also express GPR32 and FPR2, with T-helper (T_H_) 1 and T_H_17 cells expressing the highest levels of mRNA and protein in these T cell subsets.^143^ RvD1 attenuation of cytokine release from these pathogenic T_H_ cells occurs via GPR32 and FPR2 signaling.^143^ In naïve T_H_ cells, RvD1, RvD2, RvD5, or MaR1 reduce polarization into inflammatory T_H_1 and T_H_17 cells.^143^^,^^167^ Conversely, RvD1, RvD2, RvD5, or MaR1 promote differentiation of regulatory T-cells from naïve T cells and their activation, an effect mediated via GPR32 but not FPR2.^143^^,^^167^ The balance between T_H_17 and regulatory T cells may be mediated through miR-30e-5p downstream of RvD1 signaling.^164^ These findings suggest that RvD1, RvD2, RvD5, and MaR1 might differentially modulate CD4^+^ T-cell subsets through selective mechanisms. Naïve T_H_ and T_H_17 cells also express surface chemerin receptor 1, enabling them to respond to RvE1.^163^ RvE1 reduces T_H_17 polarization in response to IL-6 and TGF-β stimulation by inhibiting IL-17 and CD25 expression.^163^ However, the effect of RvE1 on T_H_ cells is less pronounced when T cells are activated by coculture with dendritic cells rather than direct cytokine treatment.^163^ It will be important to further examine the effects of SPM-GPCR interactions on B- and T-cells and their role(s) in the heart.^177^, ^178^, ^179^

## VII Effects of specialized proresolving mediators on nonimmune cardiac cell-types

**Fig. 7:**
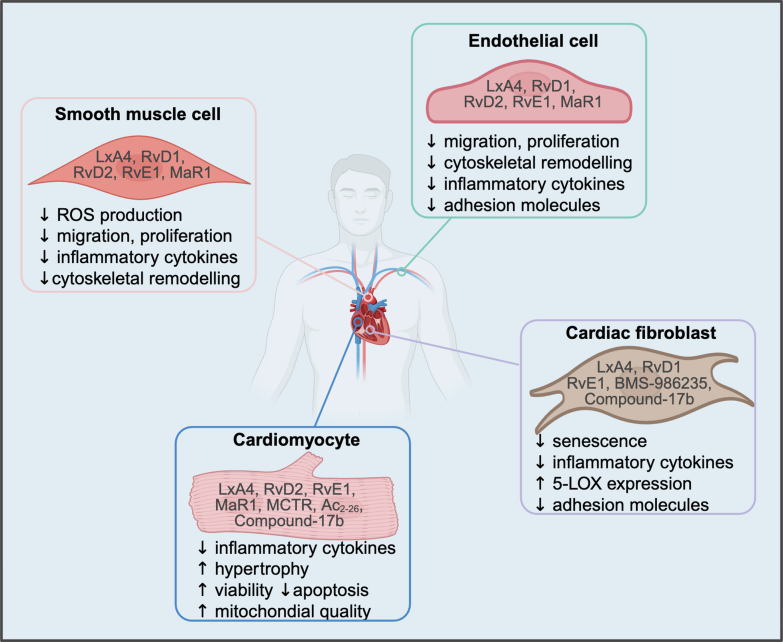
SPMs induce phenotypic changes in cell types of the heart. Cardiomyocytes, cardiac fibroblasts, smooth muscle cells, and ECs are responsive to SPM treatment. Most of these SPM-induced effects have been demonstrated in model cell lines as opposed to primary cells isolated from the heart in the context of CVD.

Activation of the innate immune system and the inflammatory response in CVD is not limited to macrophages, monocytes, and neutrophils. Nonimmune cells are also potential participants in inflammatory signaling in the heart.^180^^,^^181^ Communication between CMs, cFBs, ECs, and VSMCs, on one hand, and recruited and resident leukocytes, on the other, orchestrate the inflammatory response that influences the development of heart disease.^180^^,^^181^ CMs and cFBs secrete cytokines to act as chemoattractants for circulating leukocytes.^182^^,^^183^ Activated ECs and VSMCs upregulate expression of surface integrins that can attract and support the infiltration of immune cells.^184^ Activated VSMCs that undergo dedifferentiation take on a macrophage-like phenotype that secrete cytokines and phagocytose debris.^185^ Therefore, the role of SPM-GPCR signaling in cardiovascular nonimmune cell types also warrants investigation (Fig. 7; Table 8).^39^, ^40^, ^41^^,^^43^^,^^46^^,^^79^^,^^86^^,^^90^^,^^105^^,^^156^^,^^173^^,^^186^, ^187^, ^188^, ^189^, ^190^, ^191^, ^192^, ^193^, ^194^, ^195^, ^196^, ^197^, ^198^, ^199^, ^200^, ^201^, ^202^, ^203^, ^204^, ^205^, ^206^, ^207^, ^208^, ^209^, ^210^, ^211^, ^212^, ^213^

**Table 8:** Effects of SPM treatment in vitro on different cardiac cell-types

Cell Type	Cell Model	SPM Agonist	Phenotypic Changes	Downstream Signaling and Effectors	References
Cardiomyocyte	AVRM H-R injury	LxA4	↑ HO-1 expression, promoter activity ↓ LDH, CK release Preserved viability	p38 MAPK phosphorylation, nuclear translocation Nrf2 binding to HO-1 E1 enhancer and ARE NO Δ Akt or ERK	Chen et al 2013^186^
	H-R injury H9c2	LxA4	K_ATP_ and K_Ca_ channel activity-dependent HO-1 expression ↓ LDH, CK, TNF-α	G α_i/o_; PTX abolished effects	Chen et al 2013^43^
	AC16 Ang-II+ IFN-γ	RvD2	↓ BNP, SMA, Col1A1 gene expression	—	Diaz del Campo et al 2023^156^
	NRCM LPS	RvD2	↓ pyroptosis ↓ IL-1 β and LDH release	RvD2 effects attenuated in GSDMD deficient CMs	Zhang et al 2024^86^
	H9c2 H-R injury	RvE1	↓ caspase-3 activity, apoptosis Preserved viability	EGFR tyrosine kinase-dependent p38 MAPK, Akt, ERK1/2 phosphorylation eNOS phosphorylation	Keyes et al 2010^187^
	AC16 LPS	RvE1	↓ H-FABP leakage	—	Zheng et al 2023^188^
	NRCM	MaR1	IGF-1 production CM hypertrophy No Δ ANP or BNP expression	ROR α stimulation PI3K activation, Akt phosphorylation	Wahyuni et al 2021^189^
	H9c2 LPS	MCTR	↑ mitochondrial TFAM, PGC1 α, NRF1, NRF2 Preserved mitochondrial mass, membrane potential, respiratory complex activities, ATP content	↑ Sirt1 expression, nuclear activity	Yang et al 2020^90^
Cardiac fibroblast	Adult rat cFB High glucose	LxA4	↓ NF-κ B p-p65 phosphorylation, ICAM, MCP-1, TNF-α secretion	Akt phosphorylation ↓ JNK, p38 MAPK activation FoxO1 inhibition	Gonzalez-Herrera et al 2023^190^
	Adult murine cFB	RvD1	↑ 5-LOX expression	—	Kain et al 2019^191^
	Adult rat cFB Ang-II	RvD1	↓ IL-6 and MCP-1 secretion ↓ cFB-mononuclear cell adhesion	↓ [Ca^2+^]i	Salas-Hernandez et al 2021^192^
	Adult murine cFB Doxorubicin or IL-1 β	RvE1	↓ p53, p21, SA-β-gal, SASP profile, IL-1 β release	—	Espitia-Corredor et al 2022^193^
	Neonatal murine cFB	RvE1	No Δ matrix proteins, cytokine gene expression	—	Liu et al 2018^40^
	Neonatal rat cFB LPS	RvE1, RvD1	↓ ICAM-1, VCAM-1 expression ↓ IL-6, MCP-1, TNF-α	—	Salas-Hernandez et al 2021^41^
Endothelial cell	HUVEC VEGF or LTD_4_	LxA4	↑ FPR2 expression, IL-10 secretion ↓ proliferation, angiogenesis, stress fiber formation, ICAM-1 expression, IL-8, IL-6, TNF-α, IFN-γ secretion	↓ VEGFR-2 phosphorylation ↓ Akt, p42/44 MAPK, PLC-γ 1 activation	Baker et al 2009^194^
	Murine aortic EC TNF-α	LxA4	↓ THP1 monocyte-EC adherence ↓ MCP-1, IL-6, VCAM gene expression	—	Brennan et al 2018^195^
	PMVEC LPS	LxA4	↓ NF-κ B activation ↓ IL-1 β, IL-6, IL-8 gene expression and protein secretion	G α_i/o_; PTX abolished effects ↓ p38 and p42/44 MAPK activation, PI3K activity No Δ MyD88	Wu et al 2008^105^
	HUVEC H_2_O_2_	LxA4	↓ apoptosis, ROS, LDH, MDA, NO, PGI2 ↑ SOD levels, ET-1 Preserved viability	Nrf2-HO-1 interaction	Yang et al 2019^196^
	HUVEC TNF-α	AT-LxA4	↓ NF-κ B binding to TF promoter ↓ TF activity	↓ PI3K and Akt activation	Chen et al 2019^197^
	HUVEC VEGF	AT-LxA4	↓ stress fiber formation, actin polymerization, focal adhesion assembly, migration	G α_i/o_; PTX abolished effects ↓ p38 MAPK, FAK phosphorylation	Cezar-de-Mello et al 2006^198^
	VEGF HUVEC	AT-LxA4	Reduced, MMP-9 activity and expression, NF-κ B activation Reduced adhesion, proliferation	Reduced PI3K, ERK2 phosphorylation via PTP Reduced VEGFR-2 phosphorylation via SHP-1	Cezar-de-Mello et al 2008^199^
	HUVEC LPS	RvD1	↓ NF-κ B activity. ICAM-1 and VCAM-1 expression, THP1 monocyte-EC adhesion	G α_i/o_; PTX abolished effects ↓ PP2A phosphorylation via ↑ SHP phosphatase activity	Chattopadhyay et al 2018^200^
	HUVEC LPS	RvD1	Preserved EC barrier integrity ↓ ROS production	↓ Src kinase, Frk, phosphorylation and activation	Chattopadhyay et al 2017^201^
	HUVEC Hypoxia	RvD1	↑ angiogenesis and migration	—	Weng et al 2022^79^
	HUVEC LPS	RvD1	↓ NF-κ B activity ↓ cytoskeletal and intercellular junction rearrangement, stress fiber formation Preserved permeability barrier integrity	—	Zhang et al 2013^202^
	HUVEC TNF-α	RvD1	↓ NF-κ B activation ↓ proliferation	—	Kim et al 2022^203^
	HUVEC IL-17	RvD1, RvD2	↑ expression and secretion of anti-inflammatory Del-1	PI3K/Akt activation GSK-3 β inhibition	Maekawa et al 2015^204^
	Human microvascular EC-1 line Ang-II+ IFN-γ	RvD2	↓ NOX5, COX2, MCP-1, VCAM-1, ICAM-1, IL-6 gene expression	—	Diaz Del Campo et al 2023^156^
	MS1	RvD2	↑ migration No Δ proliferation or angiogenesis	G α_i/o_; PTX abolished effects Rac activation	Zhang et al 2016^205^
	HPAEC TGF-β 2 or IL-6	RvE1	↓α-SMA expression, EndoMT	↓ Src kinase, Fyn, and STAT3 phosphorylation and activation	Kurahara et al 2020^206^
	HUVEC Doxorubicin or IL-1 β	RvE1	↓ NLRP3 inflammasome activity ↓ p53 and p21 expression, SA-β-gal, senescence phenotype	—	Shamoon et al 2022^173^
	Murine mesenteric artery EC TNF-α	RvE1, RvD1, LxA4	↓ ROS	FPR2-independent; antagonist, WRW4, did not block effects	Edwards-Glenn et al 2023^207^
	Human saphenous vein EC TNF-α	MaR1	↓ NF-κ B activation, monocyte-EC adherence, ROS production, E-selectin, NOX2, NOX4 expression, cytokine release	↑ cAMP	Chatterjee et al 2014^208^
Vascular smooth muscle cell	Murine primary VSMC PDGF or TNF-α	LxA4	↓ NF-κ B activity, VCAM-1, IL-6, TNFRA gene expression, activation, migration, proliferation	—	Brennan et al 2018^195^
	Human pulmonary artery VSMC TNF-α or IL-6 or ET-1	RvD1	Preserved MLCP activity ↑ GPR32 expression ↓ hyperactivity	↓ CPI-17 phosphorylation	Hiram et al 2014^209^
	Rat arterial VSMC IL-1 β or PDGF	RvD1	↓ NF-κ B activation ↓ cell length to width ratio, migration, proliferation	—	Wu et al 2017^210^
	Human saphenous vein VSMC PDGF	RvD1	↓ migration rate, proliferation, stress fiber formation	—	Kim et al 2022^203^
	Human saphenous vein VSMC PDGF	AT-RvD1	↓ focal adhesion paxillin localization, cell length to width ratio, migration	↑ cAMP, PKA activity, VASP phosphorylation ↓ Rac activation	Mottola et al 2017^211^
	Murine aortic VSMC PDGF or TNF-α	RvD2, MaR1	↓ migration, proliferation, ROS production, MCP-1, NF-κ B activity No Δ IL-6, ICAM or VCAM	—	Akagi et al 2015^212^
	Human aortic VSMC Ang-II+ IFN-γ	RvD2	No Δ inflammation, adhesion, fibrosis genes	—	Diaz Del Campo et al 2023^156^
	Murine aortic VSMC High phosphate media + BMP-2	RvE1	↓ BMP-2 mRNA ↓ calcification	—	Carrecedo et al 2019^39^
	Human pulmonary artery SMC TGF-β 2 or IL-6	RvE1	↓ proliferation	↓ Src kinase activation	Kurahara et al 2020^206^
	Murine aortic VSMC PDGF	RvE1	↓ RANTES cytokine production ↓ migration No Δ proliferation	—	Liu et al 2018^46^
	Human pulmonary artery VSMC TNF-α + IL-6	RvE1, RvD1	↓ NF-κ B activation ↓ VEGF, MMP-9 ↓ migration rate	↓ nuclear c-Fos and c-Jun phosphorylation	Hiram et al 2015^213^
	Human saphenous vein VSMC TNF-α	MaR1	↓ NF-κ B activation, ROS production, NOX2 and NOX4 expression, cytokine release	↑ cAMP	Chatterjee et al 2014^208^

ANP, atrial natriuretic peptide; ARE, antioxidant response element; AVRM, adult rat ventricular cardiomyocytes; BMP-2, bone morphogenic protein 2; BNP, brain natriuretic peptide; [Ca^2+^]i, intracellular calcium concentration; CK, creatinine kinase; Col1A1, collagen 1A1; COX, cyclooxygenase; CPI-17, protein kinase C potentiated inhibitor protein 17; EGFR, epidermal growth factor receptor; EndoMT, endothelial to mesenchymal transition; eNOS, endothelial nitric oxide synthase; ET-1, endothelin-1; FAK, focal adhesion kinase; FoxO1, forkhead box protein O1; GSDMD, gasdermin D; GSK-3 β, glycogen synthase kinase-3 β; H-FABP, heart-fatty acid binding protein; H_2_O_2_, hydrogen peroxide; HPAEC, human pulmonary artery endothelial cell; H-R, hypoxia-reoxygenation; HUVEC, human umbilical vascular endothelial cell; IFN-γ, interferon gamma; IGF-1, insulin-like growth factor-1; JNK, Jun N-terminal kinase; K_ATP_, ATP-sensitive potassium channel; K_Ca_, calcium-activated potassium channel; LDH, lactate dehydrogenase; LTD_4_, leukotriene D_4_; MCP-1, monocyte chemoattractant protein-1; MDA, malondialdehyde; MLCP, myosin light chain phosphatase; MMP-9, matrix metalloproteinase-9; MS1, Mile Sven 1; MyD88, myeloid differentiation primary response 88; NLRP3, nucleotide-binding and oligomerization domain-like receptor family pyrin domain-containing 3; NO, nitric oxide; NRCM, neonatal rat cardiomyocyte; NRF1/2, nuclear respiratory factor 1/2; Nrf2, nuclear factor erythroid 2-related factor 2; PGC1 α, PDGF, platelet-derived growth factor; PGI_2_, prostaglandin I_2_; PKA, protein kinase A; PLC, phospholipase C; PMVEC, pulmonary microvascular endothelial cell; PP2A, serine/threonine protein phosphatase 2A; PTP, protein tyrosine phosphatase; Rac, Rac GTPase; RANTES, regulated on activation normal T cell expressed and secreted; ROR α, retinoic acid-related orphan receptor α; SA-β-gal, senescence-associated β-galactosidase activity; SASP, senescence-associated secretory phenotype; SHP-1, Src homology 2 domain-containing protein tyrosine phosphatase; Sirt1, sirtuin 1; SMA, smooth muscle actin; STAT3, signal transducer and activator of transcription 3; TF, tissue factor; TFAM, mitochondrial transcription factor A; THP-1, Tohoku Hospital Pediatrics-1; TNFRA, TNF receptor-α; VASP, vasodilator stimulated phosphoprotein; VEGF, vascular endothelial growth factor; VEGFR-2, vascular endothelial growth factor receptor-2; —, no information; ↑, increase; ↓, decrease; Δ, change.

### A Cardiomyocytes

Cardiomyocytes are key players in the local cardiac inflammatory response.^180^ In hypoxia-reoxygenation injury, CMs produce ROS, secrete inflammatory interleukins, and upregulate proapoptotic proteins such as cleaved caspase-3.^214^^,^^215^ Damage to CMs augments release of damage associated molecular patterns, which promote CM mitochondrial dysfunction that amplifies inflammatory injury and leukocyte recruitment. Pretreatment with LxA4 preserves CM viability, reduces apoptosis, and attenuates the release of lactate dehydrogenase and creatine kinase.^43^^,^^186^ LxA4 protection was completely abolished when cells were transfected with short interfering RNA targeting heme oxygenase-1 (HO-1).^43^ PTX treatment attenuates LxA4-induced HO-1 upregulation, which is regained with administration of K^+^-channel openers.^43^ Protein kinase A activity regulates K_ATP_-channel activity,^216^ suggesting that G α_i/o_-induced changes in protein kinase A activation might lead to downstream K^+^-channel activation and HO-1 upregulation in CMs as a protective mechanism.^43^ Compound-17b reduces cardiac troponin I release from ischemic neonatal rat and mouse CMs in vitro, possibly because of a reduction in Ca^2+^ mobilization.^68^

In adult rat ventricular CMs, LxA4-induced HO-1 upregulation is independent of Akt and ERK1/2 activation, but does require p38 MAPK phosphorylation for DNA binding of nuclear factor erythroid 2-related factor 2 to enhance HO-1 gene transcription.^186^ In H9c2 cells (a rat cardiomyocyte cell line), RvE1 increases Akt and ERK1/2 activation via the tyrosine kinase receptor, epidermal growth factor receptor, to attenuate caspase-3 activation.^187^ However, the necessity of Akt and ERK1/2 for RvE1-mediated hypoxia-reoxygenation protection is uncertain, as the impact of Akt and ERK1/2 inhibition were not examined.^187^

Effects of direct inflammatory stimulation of CMs with interferon gamma or LPS are reduced with MCTR or RvD2 treatment.^90^^,^^156^ MCTR enhances sirtuin 1 activity to preserve mitochondrial function and biogenesis, which maintains ATP production.^90^ Pretreatment of H9c2 CMs with Ac_2-26_ before LPS stimulation reduces apoptosis, along with PI3K/Akt and NF-κ B expression and caspase-3 and caspase-8 activities.^99^ MaR1 treatment of CMs induces a form of nonpathological CM hypertrophy dependent on insulin-like growth factor 1, without atrial or brain natriuretic peptide elevation.^189^ This is an intriguing mechanism, because in addition to inflammation resolution, MaR1 may stimulate cardiac repair and growth after injury.

### B Cardiac fibroblasts

Cardiac FBs, among the most abundant cells in the heart, are also involved in inflammatory signaling and communication with innate immune cells.^184^^,^^217^, ^218^, ^219^ Differentiation of quiescent cFBs into activated myofibroblasts by factors such as TGF-β or Ang-II is central to pathological remodeling and extracellular matrix deposition in heart disease.^220^ The integrin-binding molecules, ICAM-1 and VCAM-1, which are upregulated on activated cFBs, enhance proliferation and attract leukocytes.^220^ LxA4 and RvD1 reduce the expression of ICAM-1 and VCAM-1, as well as the release of proinflammatory cytokines, in cFBs.^41^^,^^190^^,^^192^ Compound-17b attenuates expression of IL-1 β and profibrotic genes in TGF-β-stimulated neonatal mouse cFBs.^68^ Physical association between cFBs and mononuclear cells in culture is also reduced by SPMs.^192^ However, pretreatment of LPS and interferongamma-stimulated monocytes with BMS-986235 did not reduce levels of VCAM-1, CCL2, or IL-8 from cFBs in a coculture system in which physical cell-interaction was blocked but substance-diffusion allowed via a permeable membrane.^91^ When monocytes were pretreated with IL-4, BMS-986235 was successful at reducing cFB VCAM-1 expression.^91^ However, the activation state of interacting immune cells, particularly “M2-like” polarized monocytes, may have enhanced sensitivity to proresolving molecules, which could also influence the phenotype of other adjacent cell types such as cFBs.

RvE1 does not affect neonatal mouse cFBs, possibly because of their immature phenotype.^40^ In adult mouse cFBs treated with IL-1 β or doxorubicin, RvE1 attenuates the development of a senescent cellular phenotype and nucleotide-binding and oligomerization domain-like receptor family pyrin domain-containing 3-inflammasome activation.^193^ RvD1 treatment of unstimulated adult mouse cFBs upregulates expression of 5-LOX, an enzyme responsible for the biosynthesis of lipid meditators including SPMs.^31^^,^^191^ RvD1 also influences 5-LOX localization in macrophages, with cytoplasmic 5-LOX favoring LxA4 biosynthesis.^152^

### C Endothelial cells

ECs are an important component of the heart.^221^ Although we were unable to identify studies of SPM effects on cardiac ECs, there are considerable data available on SPM effects on vascular ECs (Table 8). Several studies demonstrate that LxA4, RvD1, and RvD2 signaling in ECs may be coupled to G α_i/o_, affecting cell migration, attenuating inflammatory gene expression, and downregulating surface integrins.^105^^,^^197^^,^^198^^,^^205^ ROS generation is an important factor causing EC dysfunction, and quenching superoxide anions in ECs can reduce activation of inflammatory NF-κ B.^222^ SPMs reduce ROS production by modulating NADPH oxidase (NOX) isoforms responsible for the generation of superoxide from molecular oxygen; an action that may contribute to their inflammation-resolving properties in ECs.^156^^,^^196^^,^^201^^,^^207^^,^^208^

There are reports that LxA4 may damage ECs by enhancing ROS production, NOX activity, and concentration-dependent contraction in an FPR2-dependent manner.^223^ Thus, LxA4 risks detrimental actions in certain stress scenarios, such as in patients who are receiving aspirin after angioplasty procedures.^223^^,^^224^ Therefore, the microenvironment in which LxA4 exerts its effects may determine whether its actions are beneficial or harmful. The effects on EC proliferation are less clear and depend on the stressor. LxA4 and RvD1 reduce growth factor-induced EC activation and proliferation.^194^^,^^199^^,^^210^ Conversely, under hypoxic conditions, RvD1 induces angiogenesis.^79^ The fact that RvD1 induces opposite effects under hypoxia and normoxia may mean that ECs sense and respond to RvD1 according to their microenvironment.

Similar to effects in cFBs,^193^ RvE1 attenuates development of a senescent cellular phenotype in ECs exposed to doxorubicin or IL-1 β.^225^ The effects of RvD1 or LxA4 on cardiac-cell senescence need to be investigated to determine whether senescence prevention is specific to RvE1 or is also induced by other SPMs. SPM effects on other unique downstream effectors have been investigated in ECs. For example, the activity of Src-kinase family proteins, which play a role in vascular permeability, is reduced by RvE1 or RvD1 in activated ECs.^200^^,^^206^ SPM reduction of Src kinase and focal adhesion kinase activity may contribute to the preservation of the endothelial barrier and limit cytoskeletal rearrangements in ECs, reducing the infiltration of leukocytes from the blood into tissues.^194^^,^^198^^,^^201^^,^^202^^,^^206^

### D Vascular smooth muscle cells

Similar to ECs, VSMCs are important components of the heart and it is therefore relevant to consider SPM effects reported on VSMCs (Table 8).^221^ SPMs reduce inflammatory gene and cytokine expression as well as ROS levels via NOX2 and NOX4 inhibition in VSMCs cultured in the presence of inflammatory cytokines or activating growth factors.^46^^,^^195^^,^^208^^,^^212^ RvD1 signaling in human saphenous vein VSMCs may be mediated via G α_s_, as RvD1 increases cAMP and protein kinase A; whether this action occurs through FPR2, GPR32, or another unknown RvD1 receptor is not known.^211^ RvE1-chemerin receptor 1 interactions in VSMCs reduce susceptibility to calcification and maintain the contractile phenotype when cells are cultured under high phosphate conditions.^39^

The SPM-induced molecular changes in VSMCs culminate in an altered functional state. VSMCs take on a less activated phenotype with a reduction in migration rate, proliferation capacity, and cytoskeletal rearrangement.^46^^,^^195^^,^^203^^,^^206^^,^^211^, ^212^, ^213^ VSMCs are also influenced by SPM-GPCR signaling indirectly by effects on other cell types in their microenvironment. Although TNF-α-induced activation of VSMCs is unaffected by treatment with RvD2 and MaR1, pretreatment of TNF-α activated macrophages with the same SPM combination reduces collagen secretion from VSMCs that are cultured with the conditioned media from the macrophages.^159^ Future cell studies will need to take into account the diversity of the cellular environment to understand how SPMs exert their effects on immune and nonimmune cardiac cells, both directly and indirectly.

## VIII Future directions in proresolution research in cardiovascular disease

**Fig. 8:**
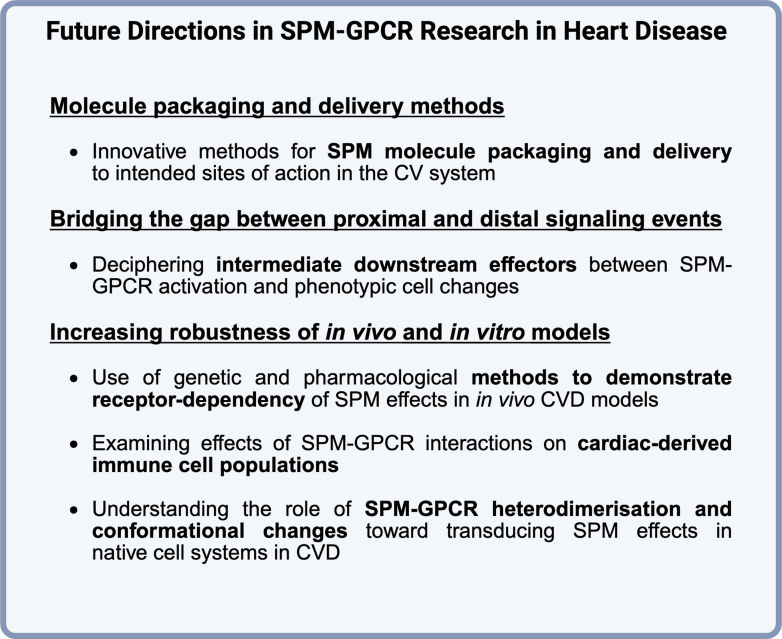
Suggested future directions for SPM-GPCR research in heart disease.

Figure 8 illustrates potential future directions for SPM-GPCR research in heart disease, which are discussed in detail below.

### A Specialized proresolving mediator packaging and delivery

Innovative delivery methods are also being developed to improve the delivery and stability of SPMs. Encapsulation into lipoproteins or nanoparticles is being used to ensure that SPMs reach the intended site of action at a therapeutically relevant concentration.^79^^,^^226^ Nanoparticles targeted to collagen IV improve delivery of the FPR2 agonist, Ac_2-26_, to atherosclerotic lesions in mice.^226^ RvD1 has been encapsulated into liposomes that mimic platelet membranes to target macrophages and monocytes recruited to the damaged heart after MI.^79^ Furthermore, these liposomes release RvD1 in response to ROS, hijacking the ROS-rich environment of the infarcted heart to ensure maximal drug delivery to the site of injury.^79^

### B Bridging the gap between proximal and distal signaling events

Efforts have been made to identify downstream signaling events of SPM-GPCR interactions.^127^ A number of common effectors have been identified, including the activation of PI3K/Akt (more proximal) and inhibition of NF-κ B transcriptional activity (distal).^38^^,^^44^^,^^55^^,^^150^^,^^189^^,^^190^^,^^195^^,^^197^^,^^199^^,^^200^^,^^202^, ^203^, ^204^^,^^208^^,^^210^ However, intermediate downstream signaling events between these proximal and distal effectors are poorly defined with respect to SPM-GPCRs in various cell types. Furthermore, there remain discrepancies between the activation or inhibition of other downstream effectors by SPMs such as MAPKs, including p38, and ERK1/2.^127^, ^128^, ^129^, ^130^ The involvement of MAPKs in orchestrating intracellular events is complex. Because the same SPM-GPCR interaction can increase and decrease MAPK activity depending on the actual situation,^127^, ^128^, ^129^, ^130^ it will be important to map out these signaling networks, taking the biological context of the SPM-GPCR interaction into consideration.

### C Increasing robustness of in vivo and in vitro models

A weakness with current in vivo studies is the lack of investigation of GPCR-dependency in the mediation of the effects of SPMs. Most studies do not include use of genetic SPM-GPCR knockout animals or coadministration of highly selective pharmacological SPM-GPCR antagonists in conjugation with SPMs to validate receptor-dependent mechanisms of observed cardioprotective effects. A handful of in vivo CVD studies have attempted to use global FPR2 knockout mice or the systemic administration of SPM-GPCR inhibitors to ascertain the receptor-dependency of SPM protective effects.^84^^,^^151^^,^^227^ However, this type of work is still in its infancy. Inducible and cell-specific receptor knockdown models would be highly useful to narrow down the cell types mediating SPM-GPCR effects in vivo and might allow for receptor knockdown at a particular period during the lifespan to eliminate off-target consequences of GPCR deficiency throughout the lifespan.^228^

CVD disproportionally affects older individuals and outcomes can be drastically different between males and females.^229^ In vivo studies of SPMs in cardiac disease have, so far, only used young animals equivalent to humans aged 20–30 years. The immune system changes with aging, complicating understanding of its interaction with the CV system.^230^ Additionally, a vast majority of studies only use male animals. Understanding the effects of aging and biological sex (male or female) is important for effective clinical translation.

SPMs have both direct and indirect effects on nonimmune cells of the CV system (Table 8). Most studies have thus far used immortalized CM cell lines, such as H9c2 cells, or neonatal CMs. These cells may not respond and signal in the same way as adult primary CMs.^231^ Future research will need to assess SPM-GPCR interactions in these more physiologically relevant cell types, ideally under conditions more representative of disease, such as cells isolated from the diseased milieu after induction of an in vivo pathology. For immune cell studies, peritoneal macrophages or bone marrow-derived macrophages, which are activated in vitro with an inflammatory stimulus such as LPS, are commonly used. Very little work has been performed to examine SPM actions on cardiac-derived immune cell populations. The study of macrophages directly isolated from diseased hearts will be important to understand SPM effects in pathophysiological states more representative of clinical heart disease.

There is also limited information on *how* SPMs bind to their cognate GPCRs on these cells. Pharmacological parameters such as *K*_*d*_ and EC_50_ have been determined after heterologous GPCR overexpression in immortalized cell lines. The complexity of SPM-GPCR heterodimerization and conformational changes, which can influence signal transduction, cannot be adequately examined in overexpression systems as they might in primary cell lines with native receptor populations and under cardiac disease-specific conditions.^48^^,^^147^

## IX Conclusions

Current evidence demonstrates a plethora of effects, mostly protective, resulting from SPM treatment in models of heart disease. However, major knowledge gaps exist, particularly concerning details of the cellular signaling networks downstream of SPM-GPCRs in cardiac disease. The development of more stable, specific, synthetic analogs of lipid-derived SPMs and their assessment in physiologically relevant cell types and animal models is necessary to advance our understanding of inflammation resolution in the heart. With the scientific interest in “resolution pharmacology” continuing to flourish, rigorous mechanistic work is crucial to move inflammation resolution from a potential target to clinically relevant therapeutics for patients with heart disease.

## Conflict of interest

The authors declare no conflicts of interest.

## References

[1] Willerson J.T., Ridker P.M. (2004). Inflammation as a cardiovascular risk factor. Circulation.

[2] Adamo L., Rocha-Resende C., Prabhu S.D., Mann D.L. (2020). Reappraising the role of inflammation in heart failure. Nat Rev Cardiol.

[3] Alfaddagh A., Martin S.S., Leucker T.M. (2020). Inflammation and cardiovascular disease: from mechanisms to therapeutics. Am J Prev Cardiol.

[4] Serhan C.N. (2017). Discovery of specialized pro-resolving mediators marks the dawn of resolution physiology and pharmacology. Mol Aspects Med.

[5] Serhan C.N. (2014). Pro-resolving lipid mediators are leads for resolution physiology. Nature.

[6] Ridker P.M., Everett B.M., Pradhan A. (2019). Low-dose methotrexate for the prevention of atherosclerotic events. N Engl J Med.

[7] Ridker P.M., Everett B.M., Thuren T. (2017). Antiinflammatory therapy with canakinumab for atherosclerotic disease. N Engl J Med.

[8] Bhatt D.L., Steg P.G., Miller M. (2019). Cardiovascular risk reduction with icosapent ethyl for hypertriglyceridemia. N Engl J Med.

[9] Jain M.K., Ridker P.M. (2005). Anti-inflammatory effects of statins: clinical evidence and basic mechanisms. Nat Rev Drug Discov.

[10] Tavazzi L., Maggioni A.P., Marchioli R. (2008). Effect of n-3 polyunsaturated fatty acids in patients with chronic heart failure (the GISSI-HF trial): a randomised, double-blind, placebo-controlled trial. Lancet.

[11] Aung T., Halsey J., Kromhout D. (2018). Associations of omega-3 fatty acid supplement use with cardiovascular disease risks: meta-analysis of 10 trials involving 77 917 individuals. JAMA Cardiol.

[12] Bowman L., Mafham M., Wallendszus K. (2018). Effects of n-3 fatty acid supplements in diabetes mellitus. N Engl J Med.

[13] Bosch J., Gerstein H.C., ORIGIN Trial Investigators (2012). n-3 fatty acids and cardiovascular outcomes in patients with dysglycemia. N Engl J Med.

[14] Kromhout D., Giltay E.J., Geleijnse J.M. (2010). Alpha Omega Trial Group. n-3 fatty acids and cardiovascular events after myocardial infarction. N Engl J Med.

[15] Manson J.E., Cook N.R., Lee I.M. (2019). Marine n-3 fatty acids and prevention of cardiovascular disease and cancer. N Engl J Med.

[16] Jamieson K.L., Endo T., Darwesh A.M., Samokhvalov V., Seubert J.M. (2017). Cytochrome P450-derived eicosanoids and heart function. Pharmacol Ther.

[17] Serhan C.N., Chiang N., Van Dyke T.E. (2008). Resolving inflammation: dual anti-inflammatory and pro-resolution lipid mediators. Nat Rev Immunol.

[18] Antonioli L., Pacher P., Haskó G. (2022). Adenosine and inflammation: it’s time to (re)solve the problem. Trends Pharmacol Sci.

[19] Lyngstadaas A.V., Olsen M.V., Bair J.A. (2021). Pro-resolving mediator annexin A1 regulates intracellular Ca^2+^ and mucin secretion in cultured goblet cells suggesting a new use in inflammatory conjunctival diseases. Front Immunol.

[20] Chen J., Norling L.V., Mesa J.G. (2021). Annexin A1 attenuates cardiac diastolic dysfunction in mice with inflammatory arthritis. Proc Natl Acad Sci U S A.

[21] Chiang N., Serhan C.N. (2017). Structural elucidation and physiologic functions of specialized pro-resolving mediators and their receptors. Mol Aspects Med.

[22] Byrne L., Guiry P.J. (2024). Advances in the chemistry and biology of specialised pro-resolving mediators (SPMs). Molecules.

[23] Dyall S.C., Balas L., Bazan N.G. (2022). Polyunsaturated fatty acids and fatty acid-derived lipid mediators: recent advances in the understanding of their biosynthesis, structures, and functions. Prog Lipid Res.

[24] Mandon E.C., de Gómez Dumm I.N., de Alaníz M.J., Marra C.A., Brenner R.R. (1987). ACTH depresses delta 6 and delta 5 desaturation activity in rat adrenal gland and liver. J Lipid Res.

[25] Bordoni A., Lopez-Jimenez J.A., Spanò C., Biagi P., Horrobin D.F., Hrelia S. (1996). Metabolism of linoleic and α-linolenic acids in cultured cardiomyocytes: effect of different N-6 and N-3 fatty acid supplementation. Mol Cell Biochem.

[26] DeLany J.P., Windhauser M.M., Champagne C.M., Bray G.A. (2000). Differential oxidation of individual dietary fatty acids in humans. Am J Clin Nutr.

[27] Norris P.C., Serhan C.N. (2018). Metabololipidomic profiling of functional immunoresolvent clusters and eicosanoids in mammalian tissues. Biochem Biophys Res Commun.

[28] Díaz Del Campo L.S., Rodrigues-Díez R., Salaices M., Briones A.M., García-Redondo A.B. (2022). Specialized pro-resolving lipid mediators: new therapeutic approaches for vascular remodeling. Int J Mol Sci.

[29] O’Donnell V.B., Schebb N.H., Milne G.L. (2023). Failure to apply standard limit-of-detection or limit-of-quantitation criteria to specialized pro-resolving mediator analysis incorrectly characterizes their presence in biological samples. Nat Commun.

[30] Schebb N.H., Kühn H., Kahnt A.S. (2022). Formation, signaling and occurrence of specialized pro-resolving lipid mediators-what is the evidence so far?. Front Pharmacol.

[31] Halade G.V., Kain V., Hossain S., Parcha V., Limdi N.A., Arora P. (2022). Arachidonate 5-lipoxygenase is essential for biosynthesis of specialized pro-resolving mediators and cardiac repair in heart failure. Am J Physiol Heart Circ Physiol.

[32] Kraft J.D., Blomgran R., Lundgaard I., Quiding-Järbrink M., Bromberg J.S., Börgeson E. (2021). Specialized pro-resolving mediators and the lymphatic system. Int J Mol Sci.

[33] Serhan C.N., Clish C.B., Brannon J., Colgan S.P., Chiang N., Gronert K. (2000). Novel functional sets of lipid-derived mediators with antiinflammatory actions generated from omega-3 fatty acids via cyclooxygenase 2-nonsteroidal antiinflammatory drugs and transcellular processing. J Exp Med.

[34] Serhan C.N., Chiang N. (2008). Endogenous pro-resolving and anti-inflammatory lipid mediators: a new pharmacologic genus. Br J Pharmacol.

[35] Fosshaug L.E., Colas R.A., Anstensrud A.K. (2019). Early increase of specialized pro-resolving lipid mediators in patients with ST-elevation myocardial infarction. EBiomedicine.

[36] Bäck M., Powell W.S., Dahlén S.E. (2014). Update on leukotriene, lipoxin and oxoeicosanoid receptors: IUPHAR review 7. Br J Pharmacol.

[37] Smrcka A.V. (2008). G protein βγ subunits: central mediators of G protein-coupled receptor signaling. Cell Mol Life Sci.

[38] Arita M., Bianchini F., Aliberti J. (2005). Stereochemical assignment, antiinflammatory properties, and receptor for the omega-3 lipid mediator resolvin E1. J Exp Med.

[39] Carracedo M., Artiach G., Witasp A. (2019). The G-protein coupled receptor ChemR23 determines smooth muscle cell phenotypic switching to enhance high phosphate-induced vascular calcification. Cardiovasc Res.

[40] Liu G., Liu Q., Shen Y. (2018). Early treatment with Resolvin E1 facilitates myocardial recovery from ischaemia in mice. Br J Pharmacol.

[41] Salas-Hernández A., Espinoza-Pérez C., Vivar R. (2021). Resolvin D1 and E1 promote resolution of inflammation in rat cardiac fibroblast in vitro. Mol Biol Rep.

[42] Zhang R., Liu S., Guo B., Chang L., Li Y. (2014). Chemerin induces insulin resistance in rat cardiomyocytes in part through the ERK1/2 signaling pathway. Pharmacology.

[43] Chen X.Q., Wu S.H., Zhou Y., Tang Y.R. (2013). Involvement of K+ channel-dependant pathways in lipoxin A4-induced protective effects on hypoxia/reoxygenation injury of cardiomyocytes. Prostaglandins Leukot Essent Fatty Acids.

[44] Ohira T., Arita M., Omori K., Recchiuti A., Van Dyke T.E., Serhan C.N. (2010). Resolvin E1 receptor activation signals phosphorylation and phagocytosis. J Biol Chem.

[45] Fredman G., Van Dyke T.E., Serhan C.N. (2010). Resolvin E1 regulates adenosine diphosphate activation of human platelets. Arterioscler Thromb Vasc Biol.

[46] Liu G., Gong Y., Zhang R. (2018). Resolvin E1 attenuates injury-induced vascular neointimal formation by inhibition of inflammatory responses and vascular smooth muscle cell migration. FASEB J.

[47] Arita M., Ohira T., Sun Y.P., Elangovan S., Chiang N., Serhan C.N. (2007). Resolvin E1 selectively interacts with leukotriene B4 receptor BLT1 and ChemR23 to regulate inflammation. J Immunol.

[48] Cooray S.N., Gobbetti T., Montero-Melendez T. (2013). Ligand-specific conformational change of the G-protein-coupled receptor ALX/FPR2 determines proresolving functional responses. Proc Natl Acad Sci U S A.

[49] Filep J.G. (2013). Biasing the lipoxin A4/formyl peptide receptor 2 pushes inflammatory resolution. Proc Natl Acad Sci U S A.

[50] Tylek K., Trojan E., Regulska M., Lacivita E., Leopoldo M., Basta-Kaim A. (2021). Formyl peptide receptor 2, as an important target for ligands triggering the inflammatory response regulation: a link to brain pathology. Pharmacol Rep.

[51] Vermi W., Riboldi E., Wittamer V. (2005). Role of ChemR23 in directing the migration of myeloid and plasmacytoid dendritic cells to lymphoid organs and inflamed skin. J Exp Med.

[52] Parolini S., Santoro A., Marcenaro E. (2007). The role of chemerin in the colocalization of NK and dendritic cell subsets into inflamed tissues. Blood.

[53] Dalli J., Winkler J.W., Colas R.A. (2013). Resolvin D3 and aspirin-triggered resolvin D3 are potent immunoresolvents. Chem Biol.

[54] Werz O., Gerstmeier J., Libreros S. (2018). Human macrophages differentially produce specific resolvin or leukotriene signals that depend on bacterial pathogenicity. Nat Commun.

[55] Krishnamoorthy S., Recchiuti A., Chiang N. (2010). Resolvin D1 binds human phagocytes with evidence for proresolving receptors. Proc Natl Acad Sci U S A.

[56] Chiang N., Dalli J., Colas R.A., Serhan C.N. (2015). Identification of resolvin D2 receptor mediating resolution of infections and organ protection. J Exp Med.

[57] Arnardottir H., Thul S., Pawelzik S.C. (2021). The resolvin D1 receptor GPR32 transduces inflammation resolution and atheroprotection. J Clin Invest.

[58] Markworth J.F., Brown L.A., Lim E. (2021). Metabolipidomic profiling reveals an age-related deficiency of skeletal muscle pro-resolving mediators that contributes to maladaptive tissue remodeling. Aging Cell.

[59] Félix-Soriano E., Sáinz N., Gil-Iturbe E. (2021). Changes in brown adipose tissue lipid mediator signatures with aging, obesity, and DHA supplementation in female mice. FASEB J.

[60] Barden A.E., Mas E., Croft K.D., Phillips M., Mori T.A. (2015). Specialized proresolving lipid mediators in humans with the metabolic syndrome after n-3 fatty acids and aspirin. Am J Clin Nutr.

[61] Chen Y.C., Su M.C., Chin C.H. (2019). Formyl peptide receptor 1 up-regulation and formyl peptide receptor 2/3 down-regulation of blood immune cells along with defective lipoxin A4/resolvin D1 production in obstructive sleep apnea patients. PLoS One.

[62] Engert L.C., Mullington J.M., Haack M. (2023). Prolonged experimental sleep disturbance affects the inflammatory resolution pathways in healthy humans. Brain Behav Immun.

[63] Fragiskos B., Chan A.C., Choy P.C. (1986). Competition of n-3 and n-6 polyunsaturated fatty acids in the isolated perfused rat heart. Ann Nutr Metab.

[64] Schmitz G., Ecker J. (2008). The opposing effects of n-3 and n-6 fatty acids. Prog Lipid Res.

[65] Valdes A.M., Ravipati S., Menni C. (2017). Association of the resolvin precursor 17-HDHA, but not D- or E- series resolvins, with heat pain sensitivity and osteoarthritis pain in humans. Sci Rep.

[66] Kirpotina L.N., Khlebnikov A.I., Schepetkin I.A. (2010). Identification of novel small-molecule agonists for human formyl peptide receptors and pharmacophore models of their recognition. Mol Pharmacol.

[67] Schoeder C.T., Mahardhika A.B., Drabczyńska A., Kieć-Kononowicz K., Müller C.E. (2020). Discovery of tricyclic xanthines as agonists of the cannabinoid-activated orphan G-protein-coupled receptor GPR18. ACS Med Chem Lett.

[68] Qin C.X., May L.T., Li R. (2017). Small-molecule-biased formyl peptide receptor agonist compound 17b protects against myocardial ischaemia-reperfusion injury in mice. Nat Commun.

[69] Sogawa Y., Ohyama T., Maeda H., Hirahara K. (2011). Inhibition of neutrophil migration in mice by mouse formyl peptide receptors 1 and 2 dual agonist: indication of cross-desensitization in vivo. Immunology.

[70] Lupisella J., St-Onge S., Carrier M. (2022). Molecular mechanisms of desensitization underlying the differential effects of formyl peptide receptor 2 agonists on cardiac structure-function post myocardial infarction. ACS Pharmacol Transl Sci.

[71] García R.A., Lupisella J.A., Ito B.R. (2021). Selective FPR2 agonism promotes a proresolution macrophage phenotype and improves cardiac structure-function post myocardial infarction. JACC Basic Transl Sci.

[72] Asahina Y., Wurtz N.R., Arakawa K. (2020). Discovery of BMS-986235/LAR-1219: a potent formyl peptide receptor 2 (FPR2) selective agonist for the prevention of heart failure. J Med Chem.

[73] Chen Z., Wu Z., Huang C. (2013). Effect of lipoxin A4 on myocardial ischemia reperfusion injury following cardiac arrest in a rabbit model. Inflammation.

[74] Zhao Q., Shao L., Hu X. (2013). Lipoxin A4 preconditioning and postconditioning protect myocardial ischemia/reperfusion injury in rats. Mediators Inflamm.

[75] Gilbert K., Bernier J., Bourque-Riel V., Malick M., Rousseau G. (2015). Resolvin D1 reduces infarct size through a phosphoinositide 3-kinase/protein kinase B mechanism. J Cardiovasc Pharmacol.

[76] Halade G.V., Norris P.C., Kain V., Serhan C.N., Ingle K.A. (2018). Splenic leukocytes define the resolution of inflammation in heart failure. Sci Signal.

[77] Kain V., Ingle K.A., Colas R.A. (2015). Resolvin D1 activates the inflammation resolving response at splenic and ventricular site following myocardial infarction leading to improved ventricular function. J Mol Cell Cardiol.

[78] Liu R., Li Z., Wang Q. (2019). Resolvin D1 attenuates myocardial infarction in a rodent model with the participation of the HMGB1 pathway. Cardiovasc Drugs Ther.

[79] Weng X., Tan H., Huang Z. (2022). Targeted delivery and ROS-responsive release of Resolvin D1 by platelet chimeric liposome ameliorates myocardial ischemia-reperfusion injury. J Nanobiotechnology.

[80] Hiram R., Xiong F., Naud P. (2024). An inflammation resolution-promoting intervention prevents atrial fibrillation caused by left ventricular dysfunction. Cardiovasc Res.

[81] Zheng Z., Zhao M., Xu Y. (2024). Resolvin D2/GPR 18 axis ameliorates pressure overload-induced heart failure by inhibiting pro-inflammatory macrophage polarization. J Lipid Res.

[82] Fu T., Mohan M., Bose M. (2024). Lipoxin A_4_ improves cardiac remodeling and function in diabetes-associated cardiac dysfunction. Cardiovasc Diabetol.

[83] Hiram R., Xiong F., Naud P. (2021). The inflammation-resolution promoting molecule resolvin-D1 prevents atrial proarrhythmic remodelling in experimental right heart disease. Cardiovasc Res.

[84] Carrillo I., Rabelo R.A.N., Barbosa C. (2021). Aspirin-triggered resolvin D1 reduces parasitic cardiac load by decreasing inflammation in a murine model of early chronic Chagas disease. PLoS Negl Trop Dis.

[85] Wang M., Liu M., Zhang J. (2020). Resolvin D1 protects against sepsis-induced cardiac injury in mice. BioFactors.

[86] Zhang W.W., Wang S.S., Ding Y.D. (2024). Cardiac Resolvin D2 ameliorates sepsis-induced cardiomyopathy via inhibiting caspase-11/GSDMD dependent pyroptosis. Free Radic Biol Med.

[87] Chen J., Purvis G.S.D., Collotta D. (2020). RvE1 attenuates polymicrobial sepsis-induced cardiac dysfunction and enhances bacterial clearance. Front Immunol.

[88] Zhang J., Wang M., Ding W. (2020). Resolvin E1 protects against doxorubicin-induced cardiotoxicity by inhibiting oxidative stress, autophagy and apoptosis by targeting AKT/mTOR signaling. Biochem Pharmacol.

[89] Zhang J., Wang M., Ye J. (2020). The anti-inflammatory mediator resolvin E1 protects mice against lipopolysaccharide-induced heart injury. Front Pharmacol.

[90] Yang Y., Zhu Y., Xiao J. (2020). Maresin conjugates in tissue regeneration 1 prevents lipopolysaccharide-induced cardiac dysfunction through improvement of mitochondrial biogenesis and function. Biochem Pharmacol.

[91] Margraf A., Chen J., Christoforou M. (2025). Formyl-peptide receptor type 2 activation mitigates heart and lung damage in inflammatory arthritis. EMBO Mol Med.

[92] Chen J., Oggero S., Cecconello C. (2023). The annexin-A1 mimetic RTP-026 promotes acute cardioprotection through modulation of immune cell activation. Pharmacol Res.

[93] García R.A., Ito B.R., Lupisella J.A. (2019). Preservation of post-infarction cardiac structure and function via long-term oral formyl peptide receptor agonist treatment. JACC Basic Transl Sci.

[94] Gavins F.N.E., Kamal A.M., D’Amico M., Oliani S.M., Perretti M. (2005). Formyl-peptide receptor is not involved in the protection afforded by annexin 1 in murine acute myocardial infarct. FASEB J.

[95] Heo S.C., Kwon Y.W., Jang I.H. (2017). Formyl peptide receptor 2 is involved in cardiac repair after myocardial infarction through mobilization of circulating angiogenic cells. Stem Cells.

[96] La M., D’Amico M., Bandiera S. (2001). Annexin 1 peptides protect against experimental myocardial ischemia-reperfusion: analysis of their mechanism of action. FASEB J.

[97] Qin C., Buxton K.D., Pepe S. (2013). Reperfusion-induced myocardial dysfunction is prevented by endogenous annexin-A1 and its N-terminal-derived peptide Ac-ANX-A1(2–26). Br J Pharmacol.

[98] Liu P., Wang L., Wang Y. (2024). ANXA1-FPR2 axis mitigates the susceptibility to atrial fibrillation in obesity via rescuing AMPK activity in response to lipid overload. Cardiovasc Diabetol.

[99] Zhang L., Zheng Y.L., Hu R.H. (2018). Annexin A1 mimetic peptide AC2-26 inhibits sepsis-induced cardiomyocyte apoptosis through LXA4/PI3K/AKT signaling pathway. Curr Med Sci.

[100] Hecht I., Rong J., Sampaio A.L.F. (2009). A novel peptide agonist of formyl-peptide receptor-like 1 (ALX) displays anti-inflammatory and cardioprotective effects. J Pharmacol Exp Ther.

[101] Singh J., Jackson K.L., Fang H. (2024). Novel formylpeptide receptor 1/2 agonist limits hypertension-induced cardiovascular damage. Cardiovasc Res.

[102] Frangogiannis N.G. (2014). The inflammatory response in myocardial injury, repair, and remodelling. Nat Rev Cardiol.

[103] Gobbetti T., Coldewey S.M., Chen J. (2014). Nonredundant protective properties of FPR2/ALX in polymicrobial murine sepsis. Proc Natl Acad Sci U S A.

[104] Liu T., Zhang L., Joo D., Sun S.C. (2017). NF-κB signaling in inflammation. Signal Transduct Target Ther.

[105] Wu S.H., Liao P.Y., Dong L., Chen Z.Q. (2008). Signal pathway involved in inhibition by lipoxin A(4) of production of interleukins induced in endothelial cells by lipopolysaccharide. Inflamm Res.

[106] Dobrev D., Heijman J., Hiram R., Li N., Nattel S. (2023). Inflammatory signalling in atrial cardiomyocytes: a novel unifying principle in atrial fibrillation pathophysiology. Nat Rev Cardiol.

[107] Hulsmans M., Clauss S., Xiao L. (2017). Macrophages facilitate electrical conduction in the heart. Cell.

[108] Hulsmans M., Schloss M.J., Lee I.H. (2023). Recruited macrophages elicit atrial fibrillation. Science.

[109] Keefe J.A., Aguilar-Sanchez Y., Navarro-Garcia J.A. (2025). Macrophage-mediated IL-6 signaling drives ryanodine receptor-2 calcium leak in postoperative atrial fibrillation. J Clin Invest.

[110] Yuan Y., Martsch P., Chen X. (2025). Atrial cardiomyocyte-restricted cleavage of gasdermin D promotes atrial arrhythmogenesis. Eur Heart J.

[111] Tourki B., Kain V., Pullen A.B. (2020). Lack of resolution sensor drives age-related cardiometabolic and cardiorenal defects and impedes inflammation-resolution in heart failure. Mol Metab.

[112] Tourki B., Kain V., Shaikh S.R., Leroy X., Serhan C.N., Halade G.V. (2020). Deficit of resolution receptor magnifies inflammatory leukocyte directed cardiorenal and endothelial dysfunction with signs of cardiomyopathy of obesity. FASEB J.

[113] Chen R., Li J., Zhou J. (2023). Prognostic impacts of lipoxin A4 in patients with acute myocardial infarction: a prospective cohort study. Pharmacol Res.

[114] Halade G.V., Kain V., Dillion C. (2020). Race-based and sex-based differences in bioactive lipid mediators after myocardial infarction. ESC Heart Fail.

[115] Keeley E.C., Li H.J., Cogle C.R., Handberg E.M., Merz C.N.B., Pepine C.J. (2022). Specialized Proresolving Mediators in Symptomatic Women with Coronary Microvascular Dysfunction (from the Women’s ischemia Trial to Reduce Events in nonobstructive CAD [WARRIOR] Trial). Am J Cardiol.

[116] Guo S., Wu J., Zhou W. (2021). Identification and analysis of key genes associated with acute myocardial infarction by integrated bioinformatics methods. Medicine.

[117] Qian J., Gao Y., Lai Y. (2022). Single-cell RNA sequencing of peripheral blood mononuclear cells from acute myocardial infarction. Front Immunol.

[118] Sun C., Zheng W., Liang L. (2023). Acute coronary syndrome May be associated with decreased resolvin D1-to-leukotriene B4 ratio. Int Heart J.

[119] Xiao S., Kuang C. (2022). Identification of crucial genes that induce coronary atherosclerosis through endothelial cell dysfunction in AMI-identifying hub genes by WGCNA. Am J Transl Res.

[120] Chiurchiù V., Leuti A., Saracini S. (2019). Resolution of inflammation is altered in chronic heart failure and entails a dysfunctional responsiveness of T lymphocytes. FASEB J.

[121] Li J., Su H., Zhu Y., Cao Y., Ma X. (2020). ETS2 and microRNA-155 regulate the pathogenesis of heart failure through targeting and regulating GPR18 expression. Exp Ther Med.

[122] Ma D., Qin X., Zhong Z.A., Liao H., Chen P., Zhang B. (2022). Systematic analysis of myocardial immune progression in septic cardiomyopathy: immune-related mechanisms in septic cardiomyopathy. Front Cardiovasc Med.

[123] Liu L., Yu Y., Hu L.L. (2021). Potential target genes in the development of atrial fibrillation: a comprehensive bioinformatics analysis. Med Sci Monit.

[124] Shi Z., Ye S., Xiang Y. (2021). circFAT1(e2) inhibits cell apoptosis and facilitates progression in vascular smooth muscle cells through miR-298/MYB axis. Comput Math Methods Med.

[125] Barua J.D., Omit S.B.S., Rana H.K., Podder N.K., Chowdhury U.N., Rahman M.H. (2022). Bioinformatics and system biological approaches for the identification of genetic risk factors in the progression of cardiovascular disease. Cardiovasc Ther.

[126] Kraft J.D., Blomgran R., Bergström I. (2022). Lipoxins modulate neutrophil oxidative burst, integrin expression and lymphatic transmigration differentially in human health and atherosclerosis. FASEB J.

[127] Merlin J., Park J., Vandekolk T.H. (2022). Multipathway in vitro pharmacological characterization of specialized proresolving G protein-coupled receptors. Mol Pharmacol.

[128] Hanson J., Ferreirós N., Pirotte B., Geisslinger G., Offermanns S. (2013). Heterologously expressed formyl peptide receptor 2 (FPR2/ALX) does not respond to lipoxin A_4_. Biochem Pharmacol.

[129] Christophe T., Karlsson A., Rabiet M.J., Boulay F., Dahlgren C. (2002). Phagocyte activation by Trp–Lys–Tyr–Met–Val–Met, acting through FPRL1/LXA4R, is not affected by lipoxin A4. Scand J Immunol.

[130] Bae Y.S., Park J.C., He R. (2003). Differential signaling of formyl peptide receptor-like 1 by Trp–Lys–Tyr–Met–Val–Met-CONH2 or lipoxin A4 in human neutrophils. Mol Pharmacol.

[131] Planagumà A., Domenech T., Jover I. (2013). Lack of activity of 15-epi-lipoxin A_4_ on FPR2/ALX and CysLT1 receptors in interleukin-8-driven human neutrophil function. Clin Exp Immunol.

[132] Krishnamoorthy S., Recchiuti A., Chiang N., Fredman G., Serhan C.N. (2012). Resolvin D1 receptor stereoselectivity and regulation of inflammation and proresolving microRNAs. Am J Pathol.

[133] Alnouri M.W., Roquid K.A., Bonnavion R. (2024). SPMs exert anti-inflammatory and pro-resolving effects through positive allosteric modulation of the prostaglandin EP4 receptor. Proc Natl Acad Sci U S A.

[134] Chiang N., Libreros S., Norris P.C., de la Rosa X., Serhan C.N. (2019). Maresin 1 activates LGR6 receptor promoting phagocyte immunoresolvent functions. J Clin Invest.

[135] Bang S., Xie Y.K., Zhang Z.J., Wang Z., Xu Z.Z., Ji R.R. (2018). GPR37 regulates macrophage phagocytosis and resolution of inflammatory pain. J Clin Invest.

[136] Peng C., Vecchio E.A., Nguyen A.T.N. (2024). Biased receptor signalling and intracellular trafficking profiles of structurally distinct formylpeptide receptor 2 agonists. Br J Pharmacol.

[137] Bena S., Brancaleone V., Wang J.M., Perretti M., Flower R.J. (2012). Annexin A1 interaction with the FPR2/ALX receptor: identification of distinct domains and downstream associated signaling. J Biol Chem.

[138] Forsman H., Önnheim K., Andreasson E., Dahlgren C. (2011). What formyl peptide receptors, if any, are triggered by compound 43 and lipoxin A4?. Scand J Immunol.

[139] Lind S., Sundqvist M., Holmdahl R., Dahlgren C., Forsman H., Olofsson P. (2019). Functional and signaling characterization of the neutrophil FPR2 selective agonist Act-389949. Biochem Pharmacol.

[140] Zhang S., Gong H., Ge Y., Ye R.D. (2020). Biased allosteric modulation of formyl peptide receptor 2 leads to distinct receptor conformational states for pro- and anti-inflammatory signaling. Pharmacol Res.

[141] Pfeil E.M., Brands J., Merten N. (2020). Heterotrimeric G protein subunit Gαq is a master switch for Gβγ-mediated calcium mobilization by Gi-coupled GPCRs. Mol Cell.

[142] Haitina T., Fredriksson R., Foord S.M., Schiöth H.B., Gloriam D.E. (2009). The G protein-coupled receptor subset of the dog genome is more similar to that in humans than rodents. BMC Genomics.

[143] Chiurchiù V., Leuti A., Dalli J. (2016). Proresolving lipid mediators resolvin D1, resolvin D2, and maresin 1 are critical in modulating T cell responses. Sci Transl Med.

[144] Fan T., Wang W., Wang Y. (2024). PDE4 inhibitors: potential protective effects in inflammation and vascular diseases. Front Pharmacol.

[145] Saha R.N., Jana M., Pahan K. (2007). MAPK p38 regulates transcriptional activity of NF-kappaB in primary human astrocytes via acetylation of p65. J Immunol.

[146] Hodges R.R., Li D., Shatos M.A. (2017). Lipoxin A_4_ activates ALX/FPR2 receptor to regulate conjunctival goblet cell secretion. Mucosal Immunol.

[147] Chen G., Obal D. (2023). Detecting and measuring of GPCR signaling—comparison of human induced pluripotent stem cells and immortal cell lines. Front Endocrinol.

[148] Mai J., Liu W., Fang Y. (2018). The atheroprotective role of lipoxin A_4_ prevents oxLDL-induced apoptotic signaling in macrophages via JNK pathway. Atherosclerosis.

[149] Kain V., Liu F., Kozlovskaya V. (2017). Resolution agonist 15-epi-lipoxin A_4_ programs early activation of resolving phase in post-myocardial infarction healing. Sci Rep.

[150] Prieto P., Rosales-Mendoza C.E., Terrón V. (2015). Activation of autophagy in macrophages by pro-resolving lipid mediators. Autophagy.

[151] Filiberto A.C., Ladd Z., Leroy V., Su G., Elder C.T., Pruitt E.Y., Hensley S.E., Lu G., Hartman J.B., Zarrinpar A., Sharma A.K., Upchurch G.R. (2022). Resolution of inflammation via RvD1/FPR2 signaling mitigates Nox2 activation and ferroptosis of macrophages in experimental abdominal aortic aneurysms. FASEB J.

[152] Fredman G., Ozcan L., Spolitu S., Hellmann J., Spite M., Backs J., Tabas I. (2014). Resolvin D1 limits 5-lipoxygenase nuclear localization and leukotriene B4 synthesis by inhibiting a calcium-activated kinase pathway. Proc Natl Acad Sci U S A.

[153] Gerlach B.D., Marinello M., Heinz J., Rymut N., Sansbury B.E., Riley C.O., Sadhu S., Hosseini Z., Kojima Y., Tang D.D., Leeper N.J., Spite M., Barroso M., Rayner K.J., Fredman G. (2020). Resolvin D1 promotes the targeting and clearance of necroptotic cells. Cell Death Differ.

[154] Sansbury B.E., Li X., Wong B., Patsalos A., Giannakis N., Zhang M.J., Nagy L., Spite M. (2020). Myeloid ALX/FPR2 regulates vascularization following tissue injury. Proc Natl Acad Sci U S A.

[155] Chiang N., Fredman G., Backhed F., Oh S.F., Vickery T., Schmidt B.A., Serhan C.N. (2012). Infection regulates pro-resolving mediators that lower antibiotic requirements. Nature.

[156] Diaz Del Campo L.S., Garcia-Redondo A.B., Rodriguez C., Zaragoza C., Duro-Sanchez S., Palmas F., de Benito-Bueno A., Socuellamos P.G., Peraza D.A., Rodrigues-Diez R., Valenzuela C., Dalli J. (2023). Resolvin D2 Attenuates Cardiovascular Damage in Angiotensin II-Induced Hypertension. Hypertension.

[157] Ganesan R., Henkels K.M., Shah K., De La Rosa X., Libreros S., Cheemarla N.R., Serhan C.N., Gomez-Cambronero J. (2020). D-series Resolvins activate Phospholipase D in phagocytes during inflammation and resolution. FASEB J.

[158] Chiang N., Riley I.R., Dalli J., Rodriguez A.R., Spur B.W., Serhan C.N. (2018). New maresin conjugates in tissue regeneration pathway counters leukotriene D(4)-stimulated vascular responses. FASEB J.

[159] Viola J.R., Lemnitzer P., Jansen Y. (2016). Resolving lipid mediators maresin 1 and resolvin D2 prevent atheroprogression in mice. Circ Res.

[160] Norling L.V., Dalli J., Flower R.J., Serhan C.N., Perretti M. (2012). Resolvin D1 limits polymorphonuclear leukocyte recruitment to inflammatory loci: receptor-dependent actions. Arterioscler Thromb Vasc Biol.

[161] Cherpokova D., Jouvene C.C., Libreros S. (2019). Resolvin D4 attenuates the severity of pathological thrombosis in mice. Blood.

[162] Wan M., Godson C., Guiry P.J., Agerberth B., Haeggström J.Z. (2011). Leukotriene B4/antimicrobial peptide LL-37 proinflammatory circuits are mediated by BLT1 and FPR2/ALX and are counterregulated by lipoxin A4 and resolvin E1. FASEB J.

[163] Oner F., Alvarez C., Yaghmoor W. (2021). Resolvin E1 regulates Th17 function and T cell activation. Front Immunol.

[164] Cheng T., Ding S., Liu S., Li X., Tang X., Sun L. (2021). Resolvin D1 improves the Treg/Th17 imbalance in systemic lupus erythematosus through miR-30e-5p. Front Immunol.

[165] Huang L., Wu J., Cao J., Sheng X., Wang M., Cheng T. (2024). Resolvin D1 inhibits T follicular helper cell expansion in systemic lupus erythematosus. Scand J Rheumatol.

[166] Navarro-Corcuera A., Zhu Y., Ma F. (2025). Resolvin D1-mediated cellular crosstalk protects against MASH. JHEP Rep.

[167] Yamada H., Saegusa J., Sendo S. (2021). Effect of resolvin D5 on T cell differentiation and osteoclastogenesis analyzed by lipid mediator profiling in the experimental arthritis. Sci Rep.

[168] Ramon S., Bancos S., Serhan C.N., Phipps R.P. (2014). Lipoxin A_4_ modulates adaptive immunity by decreasing memory B-cell responses via an ALX/FPR2-dependent mechanism. Eur J Immunol.

[169] Ramon S., Gao F., Serhan C.N., Phipps R.P. (2012). Specialized proresolving mediators enhance human B cell differentiation to antibody-secreting cells. J Immunol.

[170] Lannan K.L., Spinelli S.L., Blumberg N., Phipps R.P. (2017). Maresin 1 induces a novel pro-resolving phenotype in human platelets. J Thromb Haemost.

[171] Dufton N., Hannon R., Brancaleone V. (2010). Anti-inflammatory role of the murine formyl-peptide receptor 2: ligand-specific effects on leukocyte responses and experimental inflammation. J Immunol.

[172] Sogawa Y., Ohyama T., Maeda H., Hirahara K. (2011). Formyl peptide receptor 1 and 2 dual agonist inhibits human neutrophil chemotaxis by the induction of chemoattractant receptor cross-desensitization. J Pharmacol Sci.

[173] Drechsler M., de Jong R., Rossaint J. (2015). Annexin A1 counteracts chemokine-induced arterial myeloid cell recruitment. Circ Res.

[174] Nguyen-Chi M., Luz-Crawford P., Balas L. (2020). Pro-resolving mediator protectin D1 promotes epimorphic regeneration by controlling immune cell function in vertebrates. Br J Pharmacol.

[175] Mosser D.M., Hamidzadeh K., Goncalves R. (2021). Macrophages and the maintenance of homeostasis. Cell Mol Immunol.

[176] Leblond A.L., Klinkert K., Martin K. (2015). Systemic and cardiac depletion of M2 macrophage through CSF-1R signaling inhibition alters cardiac function post myocardial infarction. PLoS One.

[177] Ngwenyama N., Kirabo A., Aronovitz M. (2021). Isolevuglandin-modified cardiac proteins drive CD4+ T-cell activation in the heart and promote cardiac dysfunction. Circulation.

[178] Jiao J., He S., Wang Y. (2021). Regulatory B cells improve ventricular remodeling after myocardial infarction by modulating monocyte migration. Basic Res Cardiol.

[179] Sun Y., Pinto C., Camus S. (2022). Splenic marginal Zone B lymphocytes regulate cardiac remodeling after acute myocardial infarction in mice. J Am Coll Cardiol.

[180] Ninh V.K., Brown J.H. (2021). The contribution of the cardiomyocyte to tissue inflammation in cardiomyopathies. Curr Opin Physiol.

[181] Psarras S., Beis D., Nikouli S., Tsikitis M., Capetanaki Y. (2019). Three in a box: understanding cardiomyocyte, fibroblast, and innate immune cell interactions to orchestrate cardiac repair processes. Front Cardiovasc Med.

[182] Li Z., Nguyen T.T., Valaperti A. (2021). Human cardiac fibroblasts produce pro-inflammatory cytokines upon TLRs and RLRs stimulation. Mol Cell Biochem.

[183] Anzai A., Choi J.L., He S. (2017). The infarcted myocardium solicits GM-CSF for the detrimental oversupply of inflammatory leukocytes. J Exp Med.

[184] Xuan Y., Chen C., Wen Z., Wang D.W. (2022). The roles of cardiac fibroblasts and endothelial cells in myocarditis. Front Cardiovasc Med.

[185] Cao G., Xuan X., Hu J., Zhang R., Jin H., Dong H. (2022). How vascular smooth muscle cell phenotype switching contributes to vascular disease. Cell Commun Signal.

[186] Chen X.Q., Wu S.H., Zhou Y., Tang Y.R. (2013). Lipoxin A4-induced heme oxygenase-1 protects cardiomyocytes against hypoxia/reoxygenation injury via p38 MAPK activation and Nrf2/ARE complex. PLoS One.

[187] Keyes K.T., Ye Y., Lin Y. (2010). Resolvin E1 protects the rat heart against reperfusion injury. Am J Physiol Heart Circ Physiol.

[188] Zheng A., Huang N., Bean D. (2023). Resolvin E1 heals injured cardiomyocytes: therapeutic implications and H-FABP as a readout for cardiovascular disease & systemic inflammation. Prostaglandins Leukot Essent Fatty Acids.

[189] Wahyuni T., Kobayashi A., Tanaka S. (2021). Maresin-1 induces cardiomyocyte hypertrophy through IGF-1 paracrine pathway. Am J Physiol Cell Physiol.

[190] González-Herrera F., Anfossi R., Catalán M. (2023). Lipoxin A4 prevents high glucose-induced inflammatory response in cardiac fibroblast through FOXO1 inhibition. Cell Signal.

[191] Kain V., Halade G.V. (2019). Immune responsive resolvin D1 programs peritoneal macrophages and cardiac fibroblast phenotypes in diversified metabolic microenvironment. J Cell Physiol.

[192] Salas-Hernández A., Ruz-Cortés F., Bruggendieck F. (2021). Resolvin D1 reduces expression and secretion of cytokines and monocyte adhesion triggered by angiotensin II, in rat cardiac fibroblasts. Biomed Pharmacother.

[193] Espitia-Corredor J.A., Shamoon L., Olivares-Silva F. (2022). Resolvin E1 attenuates doxorubicin-induced cardiac fibroblast senescence: a key role for IL-1β. Biochim Biophys Acta Mol Basis Dis.

[194] Baker N., O’Meara S.J., Scannell M., Maderna P., Godson C. (2009). Lipoxin A4: anti-inflammatory and anti-angiogenic impact on endothelial cells. J Immunol.

[195] Brennan E.P., Mohan M., McClelland A. (2018). Lipoxins protect against inflammation in diabetes-associated atherosclerosis. Diabetes.

[196] Yang S., Zheng Y., Hou X. (2019). Lipoxin A4 restores oxidative stress-induced vascular endothelial cell injury and thrombosis-related factor expression by its receptor-mediated activation of Nrf2-HO-1 axis. Cell Signal.

[197] Chen Y., Zheng Y., Xin L. (2019). 15-epi-lipoxin A_4_ inhibits TNF-α-induced tissue factor expression via the PI3K/AKT/ NF-κB axis in human umbilical vein endothelial cells. Biomed Pharmacother.

[198] Cezar-de-Mello P.F., Nascimento-Silva V., Villela C.G., Fierro I.M. (2006). Aspirin-triggered lipoxin A4 inhibition of VEGF-induced endothelial cell migration involves actin polymerization and focal adhesion assembly. Oncogene.

[199] Cezar-de-Mello P.F., Vieira A.M., Nascimento-Silva V., Villela C.G., Barja-Fidalgo C., Fierro I.M. (2008). ATL-1, an analogue of aspirin-triggered lipoxin A4, is a potent inhibitor of several steps in angiogenesis induced by vascular endothelial growth factor. Br J Pharmacol.

[200] Chattopadhyay R., Mani A.M., Singh N.K., Rao G.N. (2018). Resolvin D1 blocks H_2_O_2_-mediated inhibitory crosstalk between SHP2 and PP2A and suppresses endothelial-monocyte interactions. Free Radic Biol Med.

[201] Chattopadhyay R., Raghavan S., Rao G.N. (2017). Resolvin D1 via prevention of ROS-mediated SHP2 inactivation protects endothelial adherens junction integrity and barrier function. Redox Biol.

[202] Zhang X., Wang T., Gui P. (2013). Resolvin D1 reverts lipopolysaccharide-induced TJ proteins disruption and the increase of cellular permeability by regulating IκBα signaling in human vascular endothelial cells. Oxid Med Cell Longev.

[203] Kim A.S., Werlin E.C., Kagaya H. (2022). 17R/S-benzo-RvD1, a synthetic resolvin D1 analogue, attenuates neointimal hyperplasia in a rat model of acute vascular injury. PLoS One.

[204] Maekawa T., Hosur K., Abe T. (2015). Antagonistic effects of IL-17 and D-resolvins on endothelial Del-1 expression through a GSK-3β-C/EBPβ pathway. Nat Commun.

[205] Zhang M.J., Sansbury B.E., Hellmann J. (2016). Resolvin D2 enhances postischemic revascularization while resolving inflammation. Circulation.

[206] Kurahara L.H., Hiraishi K., Yamamura A. (2020). Eicosapentaenoic acid ameliorates pulmonary hypertension via inhibition of tyrosine kinase Fyn. J Mol Cell Cardiol.

[207] Edwards-Glenn J.M., Fontes M.T., Waigi E.W. (2023). Specialized pro-resolving mediator improves vascular relaxation via formyl peptide receptor-2. Am J Hypertens.

[208] Chatterjee A., Sharma A., Chen M., Toy R., Mottola G., Conte M.S. (2014). The pro-resolving lipid mediator maresin 1 (MaR1) attenuates inflammatory signaling pathways in vascular smooth muscle and endothelial cells. PLoS One.

[209] Hiram R., Rizcallah E., Sirois C. (2014). Resolvin D1 reverses reactivity and Ca2+ sensitivity induced by ET-1, TNF-α, and IL-6 in the human pulmonary artery. Am J Physiol Heart Circ Physiol.

[210] Wu B., Mottola G., Chatterjee A. (2017). Perivascular delivery of resolvin D1 inhibits neointimal hyperplasia in a rat model of arterial injury. J Vasc Surg.

[211] Mottola G., Chatterjee A., Wu B., Chen M., Conte M.S. (2017). Aspirin-triggered resolvin D1 attenuates PDGF-induced vascular smooth muscle cell migration via the cyclic adenosine monophosphate/protein kinase A (cAMP/PKA) pathway. PLoS One.

[212] Akagi D., Chen M., Toy R., Chatterjee A., Conte M.S. (2015). Systemic delivery of proresolving lipid mediators resolvin D2 and maresin 1 attenuates intimal hyperplasia in mice. FASEB J.

[213] Hiram R., Rizcallah E., Marouan S. (2015). Resolvin E1 normalizes contractility, Ca2+ sensitivity and smooth muscle cell migration rate in TNF-α- and IL-6-pretreated human pulmonary arteries. Am J Physiol Lung Cell Mol Physiol.

[214] Kang P.M., Haunstetter A., Aoki H., Usheva A., Izumo S. (2000). Morphological and molecular characterization of adult cardiomyocyte apoptosis during hypoxia and reoxygenation. Circ Res.

[215] Yamauchi-Takihara K., Ihara Y., Ogata A., Yoshizaki K., Azuma J., Kishimoto T. (1995). Hypoxic stress induces cardiac myocyte-derived interleukin-6. Circulation.

[216] Lin Y.F., Jan Y.N., Jan L.Y. (2000). Regulation of ATP-sensitive potassium channel function by protein kinase A-mediated phosphorylation in transfected HEK293 cells. EMBO J.

[217] Litviňuková M., Talavera-López C., Maatz H. (2020). Cells of the adult human heart. Nature.

[218] Li L., Coarfa C., Yuan Y. (2025). Fibroblast-restricted inflammasome activation promotes atrial fibrillation and heart failure with diastolic dysfunction. JACC Basic Transl Sci.

[219] Fender A.C., Kleeschulte S., Stolte S. (2020). Thrombin receptor PAR4 drives canonical NLRP3 inflammasome signaling in the heart. Basic Res Cardiol.

[220] Kuwahara F., Kai H., Tokuda K. (2003). Roles of intercellular adhesion molecule-1 in hypertensive cardiac remodeling. Hypertension.

[221] Pinto A.R., Ilinykh A., Ivey M.J. (2016). Revisiting cardiac cellular composition. Circ Res.

[222] Nishikawa T., Edelstein D., Du X.L. (2000). Normalizing mitochondrial superoxide production blocks three pathways of hyperglycaemic damage. Nature.

[223] Wenceslau C.F., McCarthy C.G., Szasz T., Webb R.C. (2014). Lipoxin A4 mediates aortic contraction via RHOA/RHO kinase, endothelial dysfunction and reactive oxygen species. J Vasc Res.

[224] Brezinski D.A., Nesto R.W., Serhan C.N. (1992). Angioplasty triggers intracoronary leukotrienes and lipoxin A4. Impact of aspirin therapy. Circulation.

[225] Shamoon L., Espitia-Corredor J.A., Dongil P. (2022). Resolvin E1 attenuates doxorubicin-induced endothelial senescence by modulating NLRP3 inflammasome activation. Biochem Pharmacol.

[226] Fredman G., Kamaly N., Spolitu S. (2015). Targeted nanoparticles containing the proresolving peptide Ac2-26 protect against advanced atherosclerosis in hypercholesterolemic mice. Sci Transl Med.

[227] Bardin M., Pawelzik S.C., Lagrange J. (2022). The resolvin D2—GPR18 axis is expressed in human coronary atherosclerosis and transduces atheroprotection in apolipoprotein E deficient mice. Biochem Pharmacol.

[228] Doetschman T., Azhar M. (2012). Cardiac-specific inducible and conditional gene targeting in mice. Circ Res.

[229] Tsao C.W., Aday A.W., Almarzooq Z.I. (2022). Heart disease and stroke Statistics-2022 update: a report from the American Heart Association. Circulation.

[230] Liberale L., Badimon L., Montecucco F., Lüscher T.F., Libby P., Camici G.G. (2022). Inflammation, aging, and cardiovascular disease: JACC review topic of the week. J Am Coll Cardiol.

[231] Ivanova A.D., Kotova D.A., Khramova Y.V. (2024). Redox differences between rat neonatal and adult cardiomyocytes under hypoxia. Free Radic Biol Med.

