# Supplementary material for: G protein-coupled receptors and inflammation resolution signaling networks in the heart: Pharmacology and potential for innovative therapeutics
Source: Pharmacol Rev. 2026 Feb 28;78(3):100129. doi: 10.1016/j.pharmr.2026.100129 (PMC13197962; doi:10.1016/j.pharmr.2026.100129)
Supplement: Supplementary Figure 1 [file mmc1.pdf]

SUPPLEMENTAL MATERIAL

G protein-coupled receptors and inflammation resolution signaling networks in the heart: Pharmacology and potential for innovative therapeutics

Deanna K. Sosnowski, Terence E. Hébert , Dobromir Dobrev, Stanley Nattel

Corresponding author: Dr. Stanley Nattel, MD, Montreal Heart Institute Research Centre, 5000 Belanger Street Montreal, Quebec. Canada H1T 1C8

Tel: 514-376-3330 ext.: 3990 | Email: stanley.nattel@icm-mhi.org

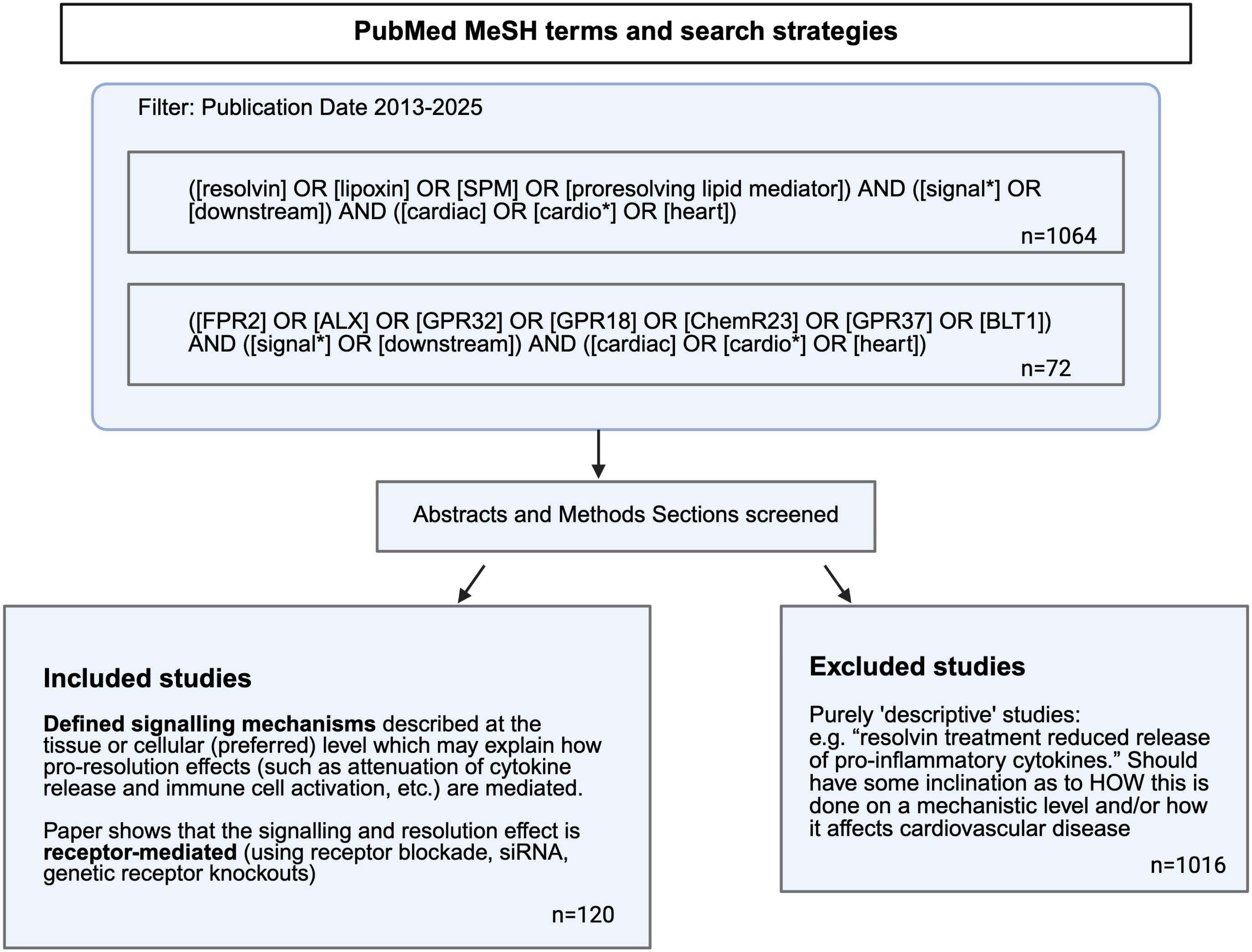

Supplemental Figure S1. Literature search and selection strategy.
